# Structure of the core human NADPH oxidase NOX2

**DOI:** 10.1038/s41467-022-33711-0

**Published:** 2022-10-14

**Authors:** Sigrid Noreng, Naruhisa Ota, Yonglian Sun, Hoangdung Ho, Matthew Johnson, Christopher P. Arthur, Kellen Schneider, Isabelle Lehoux, Christopher W. Davies, Kyle Mortara, Kit Wong, Dhaya Seshasayee, Matthieu Masureel, Jian Payandeh, Tangsheng Yi, James T. Koerber

**Affiliations:** 1 Department of Structural Biology, Genentech Inc., South San Francisco, CA 94080 USA; 2 Department of Immunology, Genentech Inc., South San Francisco, CA 94080 USA; 3 Department of Antibody Engineering, Genentech Inc., South San Francisco, CA 94080 USA; 4 Department of Biomolecular Resources, Genentech Inc., South San Francisco, CA 94080 USA; 5 DevSci OMNI-Biomarker Development, Genentech Inc., South San Francisco, CA 94080 USA; 6 Present Address: Altos Labs Inc, Redwood City, CA 94065 USA; 7 Present Address: Frontier Medicines, South San Francisco, CA 94080 USA; 8 Present Address: Expression Systems, LLC, Davies, CA 95618 USA; 9 Present Address: Exelixis Inc., Alameda, CA 94502 USA; 10 Present Address: Gilead Sciences Inc., Foster City, CA 9440 USA

**Keywords:** Immunology, Cryoelectron microscopy, Oxidoreductases

## Abstract

NOX2 is the prototypical member of the NADPH oxidase NOX superfamily and produces superoxide (O_2_^•−^), a key reactive oxygen species (ROS) that is essential in innate and adaptive immunity. Mutations that lead to deficiency in NOX2 activity correlate with increased susceptibility to bacterial and fungal infections, resulting in chronic granulomatous disease. The core of NOX2 is formed by a heterodimeric transmembrane complex composed of NOX2 (formerly gp91) and p22, but a detailed description of its structural architecture is lacking. Here, we present the structure of the human NOX2 core complex bound to a selective anti-NOX2 antibody fragment. The core complex reveals an intricate extracellular topology of NOX2, a four-transmembrane fold of the p22 subunit, and an extensive transmembrane interface which provides insights into NOX2 assembly and activation. Functional assays uncover an inhibitory activity of the 7G5 antibody mediated by internalization-dependent and internalization-independent mechanisms. Overall, our results provide insights into the NOX2 core complex architecture, disease-causing mutations, and potential avenues for selective NOX2 pharmacological modulation.

## Abstract (web summary)

NADPH oxidase NOX2 produces superoxide, a reactive oxygen species essential in innate immunity. Here, the authors reveal the structure of the NOX2 core, rationalize disease-causing mutations, and suggest avenues for selective NOX2 pharmacological modulation.

## Introduction

The reactive oxygen species (ROS) superoxide (O_2_^•−^) and hydrogen peroxide (H_2_O_2_) play important roles in cell signaling and the immune system, and are generated by enzymes belonging to the nicotinamide adenine dinucleotide phosphate (NADPH) oxidase (NOX) superfamily^1–3^. In humans, the NOX superfamily consists of seven members that differ in ROS production, subunit composition, mechanism of regulation, and tissue distribution^2,4–9^. NOX2, the most-studied member, is expressed primarily in phagocytic cells such as neutrophils and macrophages and is responsible for superoxide production during the respiratory burst, a process where ROS are rapidly generated in phagosomes to destroy microbial pathogens^10–14^. Loss-of-function mutations in NOX2 cause chronic granulomatous disease (CGD), an immunodeficiency disorder where patients develop recurrent bacterial and fungal infections^15–17^. Conversely, excessive ROS levels result in inflammation and tissue damage that can lead to diabetes, cancer, and autoimmune diseases^18^, suggesting that selective NOX2 modulation might provide therapeutic benefit.

The NOX2 core complex, historically referred to as cytochrome b_558_, is composed of two transmembrane subunits: the catalytic NOX2 subunit, historically referred to as gp91^phox^, and the auxiliary p22^phox^ (p22) subunit^2^. Activation of NOX2 occurs when four cytosolic subunits, p47^phox^ (p47), p67^phox^ (p67), p40^phox^ (p40), and Rac1 (or Rac2) are recruited to the membrane and assemble with the NOX2–p22 core complex to form the active NOX2 enzymatic complex^19–22^. This activation process is initiated by phosphorylation-dependent binding of the cytosolic p47 subunit to the C-terminus of p22, leading to recruitment of the cytosolic subunits p47, p67, p40, and Rac1 to the NOX2 core complex at the membrane^23–27^. In the activated complex, electrons are first transferred from NADPH to flavin adenine dinucleotide (FAD) bound within the intracellular domain of NOX2 and subsequently transferred via two heme molecules in the transmembrane domain of NOX2 to molecular oxygen, producing superoxide through an outer-sphere reaction^28,29^. All NOX members share a homologous NOX2-like catalytic subunit responsible for electron transfer and ROS generation^30,31^. NOX1–3 activity requires p22 and the recruitment of cytosolic subunits, whereas NOX4 only requires p22. NOX5, DUOX1 and DUOX2 do not form complexes with p22 but contain EF-hand domains involved in calcium-dependent activation^32–34^. DUOX1 and DUOX2 are the most sequence divergent family members and associate with a distinct auxiliary subunit, DUOXA1/2^33,34^.

Structures of prokaryotic NOX5 and eukaryotic DUOX1 have recently shed light on the architecture of the catalytic subunit and the electron transfer pathway^32–34^, but a detailed view of the prototypical NOX2 subfamily is lacking. Compared to NOX5 or DUOX1, NOX2 contains longer extracellular loops that are decorated with N-linked glycans, but the structural or functional consequences of these modifications remains unknown. The architecture of the p22 subunit and the mechanism by which it stabilizes the catalytic subunit is also incompletely understood. p22 has been predicted to contain two, three or four-transmembrane helices and bears low sequence identity to other proteins^35^. Missense mutations spread across both NOX2 and p22 sequences have been identified in CGD patients, but the underlying molecular causes leading to loss-of-function are not fully understood^16,17^. Pharmacologically, only small-molecule inhibitors with limited NOX selectivity are readily available, and structural details regarding NOX modulation are lacking^36^.

In this work, we elucidate the structure of the human NOX2 core complex (the NOX2–p22 heterodimer) bound to an antibody Fab (fragment–antigen binding) to provide key insights into NOX2 architecture, disease-causing mechanisms, and the potential for selective pharmacological modulation.

## Results

### Overall structure of the NOX2 core in complex with Fab 7G5

**Fig. 1:**
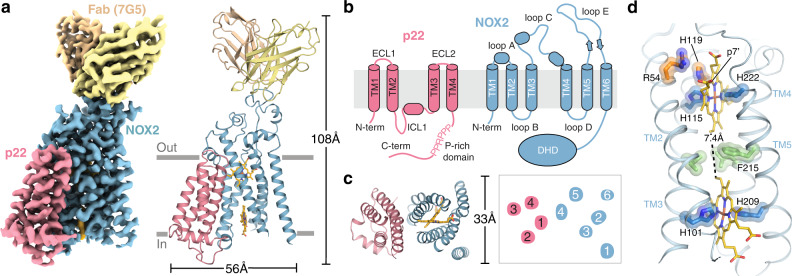
Architecture of the NOX2 core-Fab 7G5 complex. **a** Cryo-EM map (left) and three-dimensional reconstruction (right) of the NOX2 core-Fab 7G5 complex viewed parallel to the membrane plane, with approximate membrane boundaries indicated in gray. **b** Cartoon topology model of the NOX2 core complex, with the membrane bilayer colored light gray. **c** Model of the NOX2 core (left) and cartoon representation of the transmembrane helices (right), viewed perpendicular to the membrane from the extracellular side. **d** Close-up view of the NOX2 TM core, with hemes (yellow) coordinated by conserved histidine residues (blue). The metal-to-metal distance between the two hemes is 19.8 Å, with a closest interatomic distance (vinyl-to-vinyl) of 7.4 Å. The putative reduction center, composed of His115 (blue) and His119, Arg54 (orange) is located close to the p7’ propionate group of the outer heme. Hydrophobic residues located between the two heme molecules, including Phe215, are highlighted in green.

After initial efforts to structurally characterize the NOX2 core complex or larger NOX2 enzymatic complexes failed, anti-NOX2 antibodies were generated to aid in structure determination. Briefly, rabbits were immunized with extracellular vesicles containing the NOX2 enzymatic complex and boosted with purified recombinant protein reconstituted into amphipols, leading to identification of 7G5 as a promising clone for structural studies. Addition of Fab 7G5 to the NOX2 core complex purified in mild detergent significantly improved particle alignment and facilitated structure determination by cryogenic electron microscopy (cryo-EM). A three-dimensional reconstruction of the NOX2 core-7G5 Fab tripartite complex was obtained at 3.2 Å resolution (Fig. 1a, Supplementary Figs. 1 and 2, and Supplementary Table 1).

The overall NOX2 core-7G5 Fab complex resembles a vase and flower assembly, where NOX2–p22 form a composite transmembrane base while the 7G5 Fab attaches to the most membrane-distal portion of extracellular loop 3 (ECL3; or classically, extracellular loop E) on NOX2 (Fig. 1a–c). In NOX2, the heme-binding ferric oxidoreductase transmembrane domain (TMD) and all three extracellular loops (ECLs): ECL1 (loop A), ECL2 (loop C), ECL3 (loop E) are well-resolved, whereas the intracellular FAD and NADPH-binding dehydrogenase domain (DHD) is only visualized at lower cryo-EM map contours (Supplementary Fig. 2a, b, d). In p22, the TMD, both ECLs (ECL1-2) and intracellular loop 1 (ICL1) are well-resolved, whereas the intracellular C-terminus, which harbors the proline-rich region involved in p47 binding (^155^PPPRPP^160^) is disordered (Supplementary Fig. 2c, d).

### Structure of the catalytic NOX2 subunit

The TMD architecture of NOX2 closely resembles the catalytic subunits of *Cylindrospermum stagnale* NOX5 (*cs*NOX5, rmsd = 1.1 Å), mouse DUOX1 (rmsd = 0.97 Å), and human DUOX1 (rmsd = 0.95 Å)^32–34^ (Supplementary Fig. 3a), with TMs 2–5 adopting a similar hourglass-shaped scaffold that sandwiches two heme groups orthogonal to the membrane plane (Fig. 1d and Supplementary Fig. 3a)^37^. Conserved histidine residues on TM3 and TM5 coordinate the hemes (His101, His115, His209, His222), and multiple positively charged residues line the intracellular opening of the heme-binding cavity to likely facilitate electron transfer. A conserved cluster of hydrophobic residues is located in between the two hemes and may facilitate electron transfer upon activation, where Phe215 may be the preferred electron transfer path in NOX2, analogous to Trp378 in *cs*NOX5 and Phe1097 in DUOX1, respectively (Fig. 1d)^32,33^. Accordingly, the substitution of Phe215 with Ala, Val or Ile, but not Tyr, reduces NOX2 ROS production in a recombinant cell-based assay, supporting the role of an aromatic sidechain at this position in facilitating heme-to-heme electron transfer (Supplementary Fig. 4a, b). The putative oxygen reduction center in NOX2 is located between the outer heme 7-propionate group and three highly conserved positively charged residues (Fig. 1d), where an Arg54Ser mutation in CGD patients renders NOX2 non-functional^38^, identifying a key role for this residue in supporting superoxide generation.

The cryo-EM map only resolves the intracellular DHD at lower contours (Supplementary Fig. 2d), suggesting this region is dynamic in the NOX2 core complex when cytosolic subunits are absent. Structural superposition with *cs*NOX5 or DUOX1 hints at important regions involved in stabilizing the DHD, suggesting that stabilizing interdomain interactions between the TMD and DHD may be a common mechanism of activation among the NOX/DUOX members^34,39^. A lipid and/or detergent alkyl chain modeled into both *cs*NOX5^32^ and mouse DUOX1^33^ structures superimposes into a density feature present in our cryo-EM map that makes close contact with Phe100 on TM3 of NOX2 (Supplementary Fig. 3b). Phe100 is conserved in all NOX proteins (Supplementary Fig. 5) and may form part of a lipid coordination site that contributes to the stability of the NADPH-binding pocket^32,33^. In addition, structural superposition with human DUOX1 places the DHD with bound FAD of DUOX1 in close proximity to the inner heme and loop D (intracellular loop 2) of NOX2, while bound NADPH in DUOX1 is placed in close proximity to TM1 of NOX2 (Supplementary Fig. 3c, panels 2 and 3). The DUOX1 pre-TM1 helix makes electrostatic contacts with NADPH^33,34^, but pre-TM1 helix residues involved in stabilizing bound NADPH are not conserved in NOX2. Specifically, the NOX2 pre-TM1 region only consists of seven residues, and a putative pre-TM1 helix, which is not resolved in our structure, would only extend two helical turns at most (Supplementary Figs. 3a and 5). As such, we propose that binding of the cytosolic subunits p47, p67, p40, and Rac1/2 facilitates a stable interaction between the NOX2 DHD and TMD to enable activation and electron transfer.

### Extracellular cap structure of NOX2

**Fig. 2:**
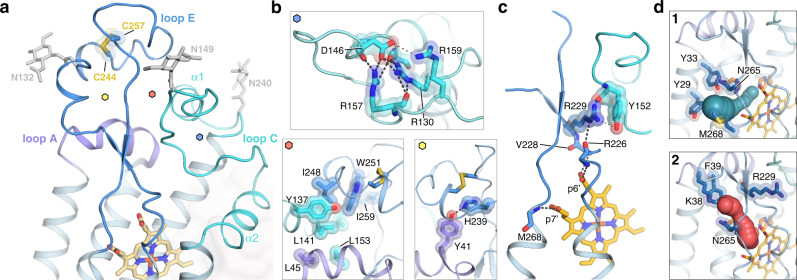
The extracellular loop cap structure of NOX2. **a** Close-up view of the NOX2 ECLs, with loops A, C, and E colored purple, cyan, and blue, respectively. The TMD of NOX2 is colored light blue, while p22 is shown as light red transparent surface. Glycans on loop C (Asn132 and Asn149) and loop E (Asn240) are represented as light gray sticks. The small hexagon labels indicate zoom-in views shown in panel **b**. **b** Close-up view of hydrophobic and polar interactions that contribute to the ECL fold, with loops and sidechains colored as in **a**. Top panel: polar intra-loop electrostatic interactions that stabilize loop C. Bottom left panel: residues that contribute to hydrophobic packing between the ECLs. Bottom right panel: electrostatic interaction between loop A residue Tyr41 and loop E residue His239, both conserved in NOX1–4. **c** Hydrogen bonds between the outer heme and the backbone of loop E residues. The p6’ propionate group contacts Arg226 and Val228, while the p7’ propionate group contacts Met268. Arg229, conserved in NOX1/3, contacts Tyr152 in loop C and connects the outer heme to the ECL A/C/E cap. **d** Putative tunnels that illustrate O_2_/O_2_^•-^ entry and exit, with putative reduction center residues colored orange. Panel 1 shows the first tunnel (green), lined by Tyr29, Tyr33 on TM1 and Asn265, Met268 on loop E. Panel 2 shows the second tunnel (red), lined by Lys38, Phe39 on loop A and Arg229, Asn265 on loop E. Sidechains that line the tunnels are represented as blue sticks.

A striking feature of the NOX2 core complex structure is that the ECLs of NOX2 fold into an organization that caps the outer heme (Fig. 2a). This extracellular cap structure of NOX2 is distinct from the ECL structures observed in *cs*NOX5 and DUOX1 (Supplementary Figs. 3a, d and  5). All three ECLs of NOX2 participate in an intricate interaction network that is anchored through numerous hydrophobic and polar interactions (Fig. 2b). Loop A (ECL1) forms a short α-helix at the base of the extracellular cap structure, which supports loop C (ECL2) and loop E (ECL3). Loop C is composed of two short α-helices that are stabilized through intra-loop electrostatic and sidechain-backbone mediated interactions (Fig. 2a, b). Loop E is the longest ECL and is composed of a short anti-parallel β-sheet, two single-turn α-helices, and a conserved disulfide bond (Cys244-Cys257). Two well-resolved N-linked glycosylation sites are found on loop C, and a third is resolved on loop E, accounting for the high glycosylation content historically observed for NOX2 (Fig. 2a).

An elaborated network of interactions in the NOX2 extracellular domain places the membrane-distal part of loop E, where the 7G5 Fab is bound, atop loop A and loop C. At the base of loop E, the outer heme within the TMD is contacted through backbone hydrogen bonds, where the Arg229 sidechain forms an interaction network connecting the outer heme to the loop C–loop E cap (Fig. 2c). Notably, loop E in *cs*NOX5 and DUOX1 are much shorter and result in different structural environments surrounding the outer heme (Supplementary Fig. 3d), whereas residues involved in forming the extracellular cap structure in NOX2 are well-conserved in NOX1–NOX4 (Supplementary Fig. 5), suggesting a potential role for these ECLs in modulating NOX function.

The program CAVER^40^ was used to define possible O_2_/O_2_^•−^ entry and exit pathways in NOX2, with His119 in the putative oxygen reduction center as the starting point. Of the four tunnels identified, the shortest one is lined by residues on TM1 and loop E, while a second tunnel forms an exit path through loop A and loop E where Lys38 may attract the negatively charged superoxide (Fig. 2d and Supplementary Fig. 6). All four tunnels constrict to a radius below 1.5 Å, consistent with an inactivate state expected for the NOX2 core complex, and indicating that loop motion or conformational dynamics are required to allow for sufficient dilation to enable O_2_/O_2_^•−^ passage in the activated state.

### Structural architecture of p22

**Fig. 3:**
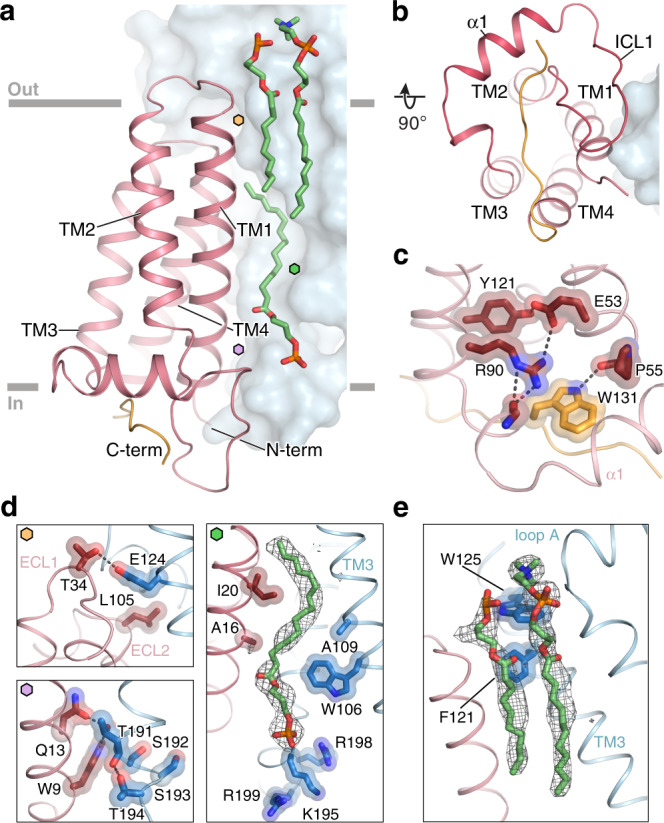
The architecture of p22 and the NOX2–p22 interface. **a** Structure model of p22 (red ribbon, with the C-terminus colored orange) associating with NOX2 (blue surface), viewed parallel to the membrane. Approximate membrane boundaries are indicated in gray and interface lipids are highlighted in green. Hexagon labels refer to zoom-in views shown in panel **d**. The buried surface area between p22 and NOX2 is ~7400 Å^2^. **b** Structure of p22 and NOX2 viewed perpendicular to the membrane. **c** Close-up view of the p22 core at the intracellular side, highlighting bulkier sidechains that contribute to hydrophobic packing and form polar interactions. The C-terminus of p22 (orange) contacts the p22 core via Trp131. **d** Close-up view of key interaction sites at the NOX2–p22 interface. Colored hexagon labels indicate the location of each site in panel (**a**). Top left: on p22 ECL1 residue Thr34 forms a hydrogen bond with NOX2 TM3 residue Glu124. The ECL2 of p22 does not extend outside the membrane and is capped by NOX2, where p22 residue Leu105 contributes to a hydrophobic patch with NOX2. Right: a lipid molecule, represented in green stick with gray mesh, is wedged between p22 and NOX2. Residues at this interface (p22:Ala16, Ile20; NOX2:Trp106, Ala109) form a shape-complementing surface for the bound lipid. Residues Lys195, Arg198, and Arg199 on NOX2 ICL2 create a positively charged patch that may favor a negatively charged phospholipid headgroup. Bottom: residues in the conserved TXXT-motif (Thr191, Ser192, Ser192, Thr194 in NOX2) form hydrogen bonds with sidechains on the N-terminus of p22 (Trp9, Gln13). **e** Two lipid molecules (green stick with gray mesh) are observed between p22 ECL1 and NOX2 loop A.

The topology of p22 has been debated, with most predictions centering on two or four-transmembrane helices, compatible with having both the N- and C-termini on the intracellular side^35^. We observe that the p22 subunit of NOX2 adopts a four-helix transmembrane fold (TM1–TM4) with two short extracellular loops connecting TM1–TM2 and TM3–TM4. The TMD core is largely embedded within the bilayer and barely extends outside the membrane on the extracellular side, where the ECLs of p22 are partially occluded by the large extracellular cap structure of NOX2 (Fig. 3a). This observation, which aligns with prior expectations^41^ and the fact that cell-surface binding antibodies raised against NOX2–p22 only target NOX2^42,43^, rationalizes why Fab 7G5 exclusively recognizes NOX2, as described below.

A long intracellular loop 1 (ICL1) in p22 connects TM2-TM3 and contains a short amphipathic helix (α1) oriented parallel to the membrane plane (Fig. 3b). The intracellular C-terminal tail is largely disordered except for the sequence proximal to TM4, which contacts the p22 core via Trp131 and positions this region close to ICL1 (Fig. 3c). These intracellular regions appear important for NOX2–p22 heterodimer stability and NOX2 function^44,45^. Prior studies have suggested that peptide fragments corresponding to p22 ICL1 and the α1 amphipathic helix can bind the cytosolic subunits p47 and p67, indicating a direct role in anchoring the cytosolic subunits to the membrane-embedded core in the activated NOX2 complex^44,46^. Structural superposition of NOX2–p22 with human DUOX1 also places p22 ICL1 residues 51–63 proximal to an α-helix in the DH domain which has been suggested to interact with p67 (Supplementary Fig. 3c, panel 1)^47,48^. In addition, the proline-rich region within the C-terminus of p22, which is known to form a key interaction with p47^49^, may become ordered upon binding of the cytosolic subunits during NOX2 activation.

The four-helical bundle of p22 widens towards the intracellular side, where bulky hydrophobic and charged residues likely contribute to its stability (Fig. 3c), while smaller sidechains are packed towards the extracellular side. The fold of p22 is simple, but no paralogs have been reported to date (Supplementary Fig. 7a). DUOX1/2 also requires an auxiliary transmembrane subunit, DUOXA1/2, for maturation and function. Notably, compared to p22, DUOXA1 adopts a different fold and binds the catalytic DUOX1 subunit at two different interfaces (Supplementary Fig. 7b, c). A DALI^50^ search of the Protein Data Base returned multiple functionally diverse four-helical bundle proteins, most of which do not exist as monomers (Supplementary Fig. 7a, d). DUOXA1 did not show up as a DALI hit, but among hits, Claudins are transmembrane proteins that assemble into homomeric structures^51^, while TARP is an auxiliary subunit involved in the regulation of AMPA receptors^52^. Thus, the p22-like four-helical bundle TMD fold appears to serve as a protein scaffold involved in diverse cellular functions.

### The NOX2–p22 interface

NOX2 and p22 assemble through an extensive, electrostatic and shape-complementary interface (∼7400 Å^2^, Fig. 3a), consistent with the obligate heterodimeric nature of the NOX2 core complex. Close inspection of the interface reveals three key interaction regions mediated primarily through TM3–TM4–TM5 of NOX2 and TM1 and TM4 of p22. The region where NOX2 interacts with p22 is analogous to where the additional M0/S0 helix of DUOX1 interacts with the DUOX1 oxidase TMD (Supplementary Fig. 3e), indicating that a potentially stabilizing interaction interface is conserved among NOX and DUOX family members. Notably, several elongated densities assigned as membrane lipids are observed across the NOX2–p22 interface that also appear to stabilize the NOX2 core complex (Fig. 3a, d, e).

The first NOX2–p22 interaction interface is located towards the extracellular side, where a polar contact connects TM3 of NOX2 with ECL1 of p22 and a hydrophobic patch connects ECL2 of p22 with NOX2 loop C (Fig. 3d). NOX1–3 conserve a negatively charged sidechain in this region, and NOX1–4 all conserve the hydrophobic patch, whereas NOX5 contains neither feature (Supplementary Fig. 5). These structural observations rationalize why the auxiliary p22 subunit would favor NOX1–4 binding over NOX5 at this region.

The second NOX2–p22 interaction interface is buried within the membrane and primarily formed by hydrophobic contacts between TM3–TM4 of NOX2 and TM1 and TM4 of p22 (Fig. 3d). The cryo-EM map reveals an elongated density that extends toward the intracellular side, suggesting that a phospholipid sandwiched in the widening interface between p22 TM1 and NOX2 TM3 may stabilize the interface. The NOX2 residues Trp106, Ala109, and Ala113 face the modeled lipid and our mutagenesis studies support a role for Trp106 and Ala113 in mediating favorable lipid packing and optimal NOX2 function (Supplementary Fig. 4c, d). Several proximal NOX2 loop D residues create a positive electrostatic charge patch that may favor the association of a negatively charged phospholipid headgroup in this region (Fig. 3d).

Sequence comparison indicates that TM4 of NOX4 contains a greater number of hydrophobic residues at the second interface than NOX1–3 (Supplementary Fig. 5). These sequence differences may rationalize the reported tighter association of NOX4 with p22 and its increased resistance to heterodimer disruption when the p22 core is perturbed^53,54^. For instance, substituting the p22 core residue Tyr121 for His abolished NOX2 and NOX3 function, but NOX4 activity was preserved^53,54^.

The third NOX2–p22 interaction interface is located on the intracellular side and mediated by multiple hydrophobic and electrostatic interactions between TM4–loop D–TM5 of NOX2 and the N-terminus and ICL1 of p22. Polar residues on p22 TM1 interact with NOX2 TM4 residues Thr191 and Thr194, sidechains that define the TXXT-motif (where either one or both X is a serine residue) which is conserved in NOX1–4 but absent in NOX5 (Fig. 3d and Supplementary Fig. 5). Deletion and mutagenesis studies indicate that the N-terminus and ICL1 of p22 as well as loop D of NOX2 are essential for NOX1–4 function^54,55^, consistent with these interactions being critical for correct assembly of the NOX2 complex.

### Structural and functional characterization of anti-NOX2 7G5

**Fig. 4:**
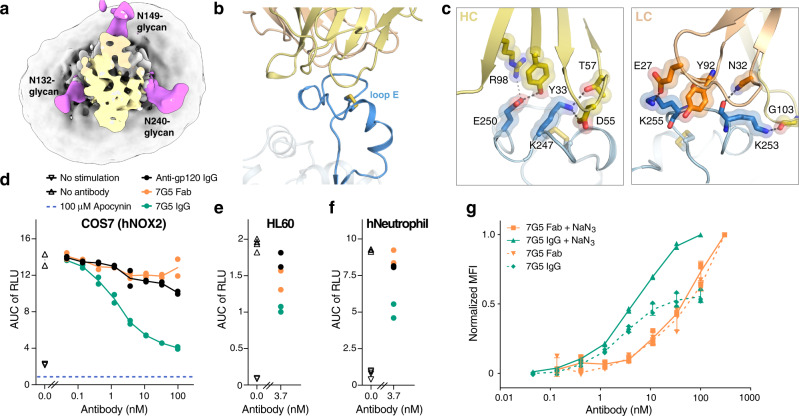
Structural and functional characterization of the anti-NOX2 antibody 7G5. **a** Cryo-EM map of the NOX2 core-Fab 7G5 complex. The Fab 7G5 (colored light orange and yellow) binds within a collar formed by three distinct glycosylation sites (colored magenta). **b** Close-up view of the NOX2-Fab 7G5 binding interface (Fab heavy chain in yellow and light chain in light orange), highlighting the small disulfide-capped epitope in loop E (blue). **c** Key interactions between NOX2 and Fab 7G5. Three lysine residues and a glutamate in NOX2 loop E form hydrogen bonds and salt bridges with negatively charged and polar residues in Fab 7G5. The left and right panels highlight key interactions between NOX2 and the heavy and light chain of 7G5, respectively. **d** Extracellular ROS production assay on COS7 cells expressing the recombinant NOX2 enzymatic complex. Dose-dependent inhibition is observed upon the addition of 7G5 IgG (green), but not with 7G5 Fab (orange). Data represent two replicates per condition with line representing the mean value. Each data point was generated by taking the area under the curve (AUC) from a ROS production assay illustrated in Supplementary Fig. 8c. The amount of ROS in the ROS production assay was measured by relative light unit (RLU). The blue dashed line represents ROS inhibition by apocynin as illustrated in Supplementary Fig. 8b. ROS production in untreated cells without and with PMA stimulation is shown by downward (four replicates) and upward (four replicates) triangles, respectively. **e**, **f** Extracellular ROS production assay on HL60 cells (**e**) and human neutrophils (**f**) expressing endogenous NOX2. The 7G5 IgG shows inhibition of ROS production while the 7G5 Fab shows weak or no inhibition. Data were generated the same way as explained in **d**. **g** The 7G5 IgG, but not the Fab, induces NOX2 internalization. Data represent the normalized mean fluorescence intensity (MFI) with three replicates per condition, and error bars represent standard deviation (SD). MFIs were normalized to the samples with sodium azide for either the IgG or Fab, respectively. Source data are available in the Source Data File.

Fab 7G5 exclusively targets the membrane-distal portion of loop E on NOX2, where the engagement interface is dictated by the distinctive extracellular cap and glycosylation structure of NOX2. Fab 7G5 binds to a short and structured segment of 12 amino acids which is constrained by a conserved disulfide bond and surrounded by a collar of three well-resolved N-linked glycans (Fig. 4a, b). Sequence conservation within this region of loop E is low, and residues in the complementary determining regions (CDRs) of 7G5 form direct interactions with three lysine sidechains that are not present in loop E of any other NOX family member. This structural analysis indicates that 7G5 is highly specific and selective for binding NOX2 over related NOX enzymes (Fig. 4c and Supplementary Fig. 8a).

Antibody antagonists targeting NOX2 have previously been described, but their molecular mechanism has remained obscure^42^. To determine if the 7G5 antibody might have a functional effect, we performed cell-based assays. NOX2 was activated by phorbol myristate acetate (PMA) treatment, and the resulting superoxide generation was measured in the presence of 7G5, using the small-molecule pan-NOX inhibitor apocynin as a control (Supplementary Fig. 8b)^7,56^. A dose-titration of NOX2-expressing COS7 cells revealed that 7G5 IgG acted as a potent partial inhibitor, but had no functional effect as a Fab (Fig. 4d and Supplementary Fig. 8c). In peripheral blood leukocyte-derived HL60 cells, where NOX2 is the main producer of extracellular superoxide (Supplementary Fig. 9a), inhibition of NOX2 activity was also observed with 7G5 IgG, with minimal inhibition observed for the Fab (Fig. 4e and Supplementary Fig. 9b, d). Experiments with freshly isolated primary human neutrophils similarly demonstrated that only the 7G5 IgG inhibited NOX2 function (Fig. 4f and Supplementary Fig. 9c, e). Cell-surface binding experiments indicated that 7G5 is competent to bind NOX2 both before and after activation (Supplementary Fig. 9f).

The relative IgG and Fab binding affinities on HL60 cells were measured, but similar values (∼7–8 nM) indicated that affinity difference is not the primary cause underlying the observed stark functional difference (Supplementary Fig. 9g). To evaluate if the 7G5 IgG and Fab differentially drive NOX2 internalization, the amount of IgG and Fab bound to NOX2 expressed on the surface of HL60 cells was measured in the absence or presence of sodium azide, which blocks internalization. Sodium azide treatment increased the cell-surface staining by about twofold for the IgG but had no effect on the Fab, indicating that internalization could contribute to IgG-mediated NOX2 inhibition (Fig. 4g). The approximate half-maximal concentration of IgG internalization (∼8 nM) is similar to the IgG cell-based affinity, but substantially higher than the apparent half-maximal concentration of NOX2 inhibition (0.12 nM, Supplementary Fig. 9h), which suggests that another undefined mechanism likely contributes to the inhibitory effect of the IgG. However, a cell-free assay of NOX2 activity on resuspended HL60 membrane fractions shows no inhibition by 7G5 IgG (Supplementary Fig. 9i), indicating that the constraint of a native cellular membrane environment combined with a high-affinity bivalent antibody is somehow responsible for the discrepancy between the half-maximal concentration of IgG internalization and NOX2 inhibition. Future studies will be required to fully elucidate the mechanism by which 7G5 IgG binding inhibits NOX2.

## Discussion

The architecture of NOX2 enzymatic complex has remained elusive despite its physiological importance. Here, we have resolved the structure of the inactive heterodimeric NOX2–p22 core complex bound to the selective anti-NOX2-Fab 7G5. The p22 subunit adopts a four-helix TMD fold that binds the catalytic NOX2 subunit across three extensive and conserved interface regions, which includes well-resolved membrane lipids, explaining the tight and requisite association between p22 and NOX2. Structure-based sequence comparison of NOX family members allows rationalization of heterodimer stability differences between NOX1–4, and the lack of p22 binding to NOX5. The extracellular loops of NOX2 are highly ordered and form a glycan-decorated cap atop the outer heme. Notably, this ECL cap appears conserved across the NOX1–4 subfamily, where residues within this region are known to be essential for NOX2 expression and function^17^. For instance, mutation of either loop E cysteine leads to a dramatic loss of ROS production in NOX1 or NOX4, identifying the conserved disulfide bond as an important structural feature^57^.

**Fig. 5:**
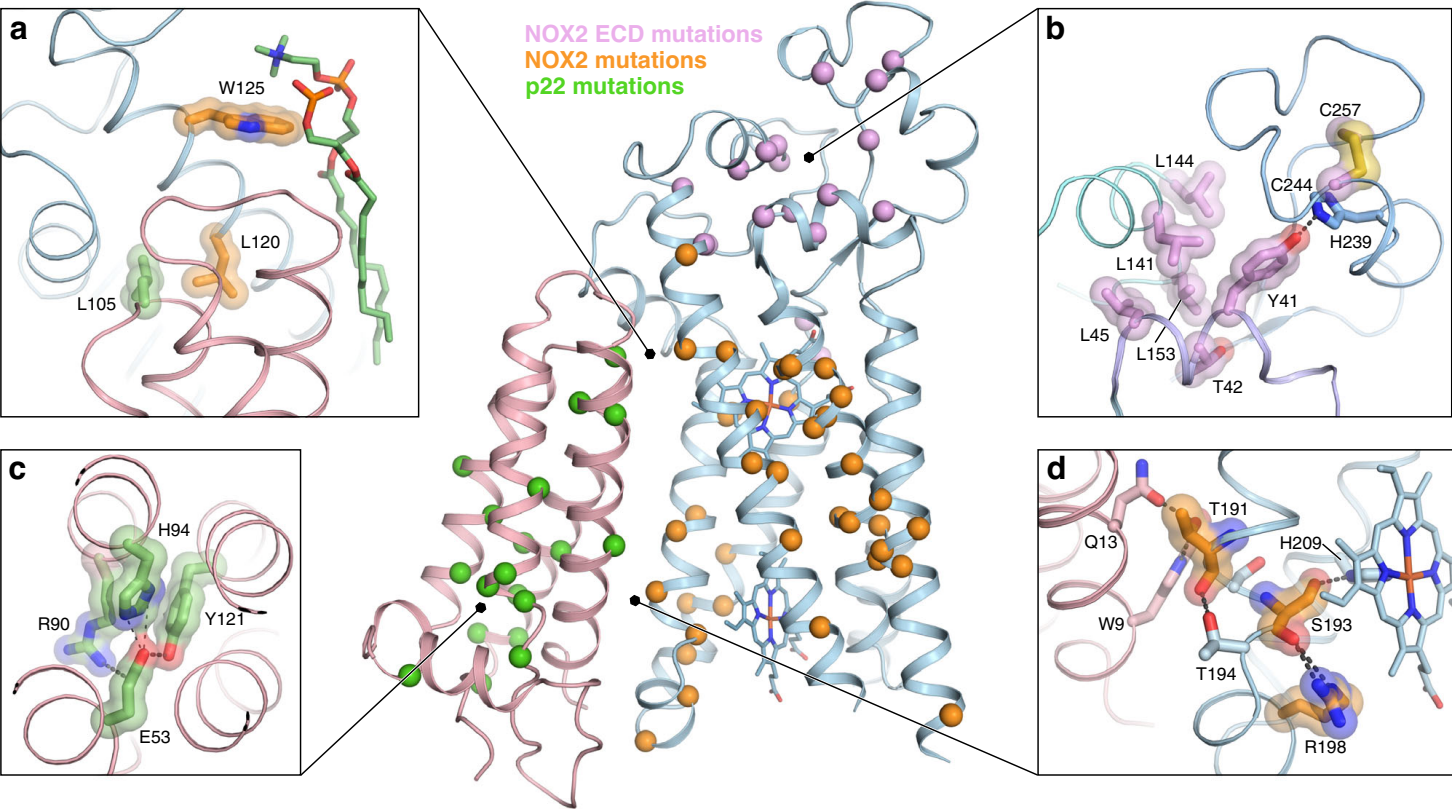
CGD mutations in the NOX2 core complex. Reported missense mutations in p22, NOX2 TMD and NOX2 ECL that lead to chronic granulomatous disease (CGD) are highlighted in green, orange, and pink, respectively. The center panel shows an overall view, with CGD mutations shown as spheres. Side panels provide a close-up view of selected mutations. Sidechains not reported as CGD mutations, but that are part of relevant interactions or the nearby chemical environment are colored light red for p22 and blue for NOX2. **a** Missense mutations Leu105Arg and Leu120Pro are predicted to disrupt the hydrophobic patch at the p22 ECL-NOX2 interface, and mutation Trp125Cys is expected to disrupt interaction with two lipids found at the interface. **b** The conserved loop E disulfide bond would be disrupted by mutations Cys244Ser/Arg/Gly/Tyr and Cys257Arg/Ser. Missense mutations Leu45Arg, Leu141Pro, Leu144Pro, and Leu153Arg are predicted to disrupt the hydrophobic packing in the ECLs, and the Tyr41Asp mutation would break an important hydrogen bond that connects loop A and E. **c** The Glu53Val/Gln, Arg90Trp/Gly/Gln/Pro, His94Arg, and Tyr121His missense mutations are predicted to destabilize the core of p22 and subsequently disrupt NOX2–p22 association. **d** Missense mutations Thr191Ser and Ser193Pro/Phe in the conserved ^191^TXXT^193^-motif and Arg198Trp, conserved in NOX1–5 and DUOX1/2, would disrupt important interactions between NOX2 and p22, potentially destabilizing the structural scaffold near the inner heme.

The impact of multiple pathogenic NOX2 mutations reported from human CGD patients can finally be understood based on the NOX2 core structure (Fig. 5 and Supplementary Table 2)^16,58,59^ (http://structure.bmc.lu.se/idbase/CYBBbase/). Among the well-documented missense mutations in p22, most map within the transmembrane core, while some reside within the disordered C-terminal region and prevent p47 association. Our structural analysis suggests that the majority of pathogenic mutations destabilize the p22 fold, which should weaken or disrupt the interaction with NOX2 (Fig. 5c). For instance, mutation of His94 to Arg is expected to disrupt the interaction with Asp53 and Tyr121 at the center of the p22 core, thereby rendering NOX2 non-functional by impacting its folding, trafficking or stability^60^. In fact, many pathogenic mutations targeting the transmembrane core of NOX2 and p22 generally appear to impact subunit folding or stability, suggesting the possibility to identify small-molecule chemical chaperones to potentially correct these defects^61^. Multiple missense mutations that lead to CGD are also found at the large interface between NOX2 and p22, in motifs close to and distal from the hemes (Fig. 5a, d), indicating that proper structural organization of the heterodimer core is crucial. Within the extracellular cap structure of NOX2, pathogenic mutations of Tyr41 and Thr42 in loop A are predicted to disrupt the stable assembly of loop E, while mutation Leu153Arg would break the hydrophobic core that stabilizes packing of loop A–loop E (Fig. 5b). In addition, mutation of Arg130 would break a key interaction within loop C (Fig. 2b), and mutation of Cys244 or Cys257 would disrupt the conserved loop E disulfide (Fig. 5b).

The NOX2 core structure, 7G5 antibody activity, and previously published results collectively suggest a mechanism by which the ECLs may allosterically modulate NOX2 function. A conserved network of interactions putatively links the propionate groups of the outer heme to the most membrane-distal part of loop E, where Arg229 may participate in linking loop E with loop C and could act as a modulatory or relay hub (Fig. 2c). Comparisons with the *cs*NOX5 and DUOX1 structures suggests that conformational changes within the NOX2 ECLs could affect interactions between the propionate groups of the outer heme and the putative oxygen reduction center to potentially regulate catalytic activity (Supplementary Fig. 3a, d). Loop E in NOX2 forms part of the apparent O_2_/O_2_^•−^ tunnel, and it therefore seems plausible that conformational changes and dynamics within this region could modulate NOX2 function by altering the ROS pathway. It is notable that 7G5 binds the most membrane-distal part of loop E and requires bivalency to inhibit NOX2, potentially through an allosteric mechanism. Bivalent binding by an IgG is known to generate steric strain between both Fab arms^62^, and this may impose a force or constraint onto the distal part of loop E sufficient to impact NOX2 activity within the context of native cell membranes. These speculations appear supported by CGD disease-causing mutations in NOX2 that infiltrate the extracellular cap observed in NOX2. The structure of the activated NOX2 complex will also undoubtedly provide further insight into activation and disease-causing mechanisms.

To date, the inhibition of NOX family members has largely been achieved by small-molecule ligands which target intracellular regions, where subtype selectivity has been difficult to achieve^36^. Demonstration that the extracellular cap of NOX2 can be targeted by a selective antibody raises the intriguing possibility of a new therapeutic strategy in NOX2-mediated pathologies. Discovery and characterization of the anti-NOX2 antibody 7G5 in combination with the structure of the NOX2 core-7G5 Fab complex adds to the growing evidence that antibody-based functional modulation of integral membrane proteins^63–65^ may be a viable pharmacological approach. Overall, our study provides a foundation to understand the structural assembly and regulation of NOX2, the pathogenesis of CGD mutations, and potential avenues for future drug discovery.

## Methods

### Expression and purification of NOX2-Fab 7G5 complex

Wild-type human NOX2 and p22^phox^ were cloned into a bicistron in a pRK vector, each with a separate CMV promoter, generated from UniProt IDs: P04839 (NOX2) and Q86SL0 (p22^phox^). A twin-Strep tag, enhanced green fluorescent protein (eGFP), and a Tobacco Etch Virus (TEV) recognition site was fused to the N-terminus of NOX2, while p22^phox^ remained untagged. Expi293 cells in suspension were cultured in SMM293T-I medium under 5% CO_2_ at 37 ^o^C until the cell density reached 4  ×  10^6^ cells per mL. Cells were then transfected using polyethylenimine and cells were cultured for 48 h before the cell pellet was harvested.

Thirty-five grams of cell pellet was resuspended and dounce-homogenized in a buffer containing 50 mM TRIS pH 8, 200 mM NaCl, Roche protease inhibitor tablets, and 1 µg/mL benzonase, and then subsequently solubilized by adding 1% (w/v) n-dodecyl-beta-D-maltopyranoside (DDM, Anatrace) for 1 h at 4 °C. The solubilized fraction was isolated by ultracentrifugation (100,000 × *g*) for 1 h at 4 ^o^C, and the resulting supernatant was subsequently filtered twice (0.22-µm cutoff) to remove insoluble material. The clarified lysate containing solubilized NOX2–p22 core complex was then affinity purified over streptactin resin packed in a gravity flow column. The column was washed with 10 column volumes (CV) of buffer A (0.03% DDM, 50 mM TRIS pH 8, 200 mM NaCl). NOX2–p22 was eluted with 5 CV of buffer A supplemented with 10 mM desthiobiotin. The eluted fraction was concentrated to 500 µL and then incubated with Fab 7G5 in a 1:3 molar ratio of NOX2–p22:Fab 7G5 for 10 min. Aggregates were removed by centrifugation-based filtration using a 0.22-µm cutoff filter, and the NOX2–p22-Fab 7G5 complex was then injected onto a Superdex 200 Increase 10/300 GL column equilibrated in 20 mM TRIS pH 8, 150 mM NaCl, 0.03% DDM. Fractions from a monodisperse peak corresponding to the complex were pooled and concentrated to 1.2 mg/mL.

### Sample preparation, cryo-EM data collection, and data processing

Purified NOX2–p22-Fab 7G5 at a concentration of 1.2 mg/mL was applied on glow-discharged holey-carbon grids (Ultrafoil 25 nm Au RO 0.6/1 300 mesh; Quantifoil). Grids were glow-discharged for 10 s on a Solarus plasma cleaner (Gatan), and then loaded on a Vitrobot Mark IV (Thermofisher) with 100% humidity and 4 °C. 3.5 µL of purified NOX2–p22-Fab 7G5 were applied on the carbon side of the grid with a wait time of 5 s, 3.5 s blot time at blot force 7, before plunge-freezing in liquid ethane cooled by liquid nitrogen.

A large data set of NOX2–p22-7G5 was collected using a Titan Krios equipped with a Falcon4 detector. The software EPU (Thermo Fisher) was used for the data collection of 17,785 movie stacks total at a pixel size of 0.731 Å. Acquisition time was 4.5 s, and movie stacks were dose-fractionated to 1041 frames with a dose rate of 0.039 e^-^/Å^2^/frame and total dose of 40.422 e^-^/Å^2^.

Movie frames were imported to cryoSPARCv3.1.0 motion corrected using Patch motion correction (multi), and defocus values were estimated using Patch CTF estimation (multi)^66^. Micrographs with defocus values higher than 5 µM and micrographs with no particles were discarded resulting in 4559 micrographs used in the data processing. Initial templates were generated from 2D classes from a smaller data set of the same sample, resulting in 833,897 picked particles. Multiple rounds of 2D classification, including 3D classification and refinement resulted in a 3D reconstruction of 4.1 Å map (GS-FSC, gold-standard Fourier shell correlation) (Supplementary Fig. 1c). The best 2D classes were used as template for automated particle picking using cryoSPARC template picker, resulting in 581,619 particles that were extracted and binned by 1.5. Multiple rounds of 3D classification, including ab initio and heterogenous refinement with 2–4 classes during each iteration were performed. For each round, the best class was assessed by non-uniform refinement and local refinement until no improvement was seen in map quality and GS-FSC. No other major classes of NOX2–p22-Fab 7G5 were observed during 2D classification, 3D classification, and 3D refinement in cryoSPARCv3. The final stack of particles of 70,486 particles were re-extracted with no binning, and local CTF refinement per particle in cryoSPARC was performed. After CTF refinement, particles were processed using local refinement in cryoSPARC with default settings and C1 symmetry. The final 3D reconstruction was reported at a resolution of 3.16 Å (GS-FSC).

### Model building

To accelerate our model-building efforts, coordinates of p22 and NOX2 were obtained from the AlphaFold2 (AF2) structure data bank^67^ and docked in the cryo-EM map using Chimera. Residue numbers 7–74 and 96–287 were fit into the cryo-EM map of NOX2, and residue numbers 4–135 were fit into the cryo-EM map of p22. All coordinates were manually inspected and adjusted using the program COOT^68^ and ISOLDE^69^ with iterative rounds of real-space refinement using PHENIX^70^. The final model was determined to have good stereochemistry and fit within cryo-EM map as assessed by MolProbity^71^ and Q-scores (MapQ)^72^. Distance measurements and figures were made using the software Pymol^73^ and ChimeraX^74^. Notably, the overall RMSD between the final NOX2 and p22 experimental models and the AF2-generated models is 0.601 Å, and 0.485 Å, respectively.

### Anti-NOX2 antibody generation

Extracellular vesicles (EVs) were generated containing the entire NOX2 complex (NOX2, p22, p47, and p67). Expi293 cells were co-transfected with mammalian expression constructs encoding for each subunit (NOX2, p22, p47, and p67) and a mammalian expression construct encoding for MLGag^75^. Seven days post-transfection, EVs were purified from the supernatant using ultracentrifugation as previously described^76^.

New Zealand White (NZW) rabbits (Charles River Laboratories, Hollister, CA) were initially immunized with NOX2 EVs in PBS followed by two injections of purified NOX2 complex in amphipols, reconstituted by established methods^77^. Animals used in these studies were maintained in an Association for Assessment and Accreditation of Laboratory Animal Care (AAALAC)-accredited animal facility. All experiments were performed in compliance with Genentech’s Institutional Animal Care and Use Committee (IACUC) and National Institutes of Health’s Office of Laboratory Animal Welfare Guidelines. Approval of the study design was obtained from the Genentech IACUC prior to the start of this work.

To generate monoclonal antibodies, up to 30 mL of blood was drawn from each immunized rabbit and peripheral blood mononuclear cells (PBMCs) were isolated by density centrifugation of whole blood over Lympholyte®-M (CL5115, Cedarlane Labs), and IgG+ B cells were enriched by staining with antibody cocktails containing 1:30 of anti-rabbit CD11b and 1:40 of anti-rabbit T-lymphocyte antibody (MCA802GA and MCA800GA respectively, AbD Serotec, BioRad), as well as 1:20 of anti-rabbit IgM (550938, BD Bioscience), and negatively selected through MACS Column (130-042-401, Miltenyi Biotec). The enriched rabbit B cells were stained with mixed APC-labeled mouse anti-rabbit IgG antibody (Purified mouse anti-rabbit IgG, Southern Biotech 4090-01) labeled with APC using Lightning-Link® APC Antibody Labeling Kit (Novus Biotech, 705-0030) and PE-labeled NOX2 enzymatic complex (labeled with Lightning-Link (R) R-PE Antibody Labeling Kit, Novus Biotech 703-0010) in the staining buffer (Phosphate-buffered saline with 0.5% bovine serum albumin and 2 mM EDTA), and single IgG+, NOX2 protein-binding B cells were sorted into 96-well plate with supplemented RPMI 1640 culture medium and EL4-B5 feeder cells for 7 days cultivation at 37 °C, as previously described^78^.

The B-cell culture supernatants were harvested and screened by ELISA for IgG production and by FACS for binding to cell-surface expressed NOX2. The variable light chain and variable heavy chain sequences from anti-NOX2 B cells were obtained by Sanger sequencing as previously described^78^.

### Antibody and Fab production

IgG and Fab expression constructs for the light chain and heavy chain for 7G5 were obtained by gene synthesis (Genscript, South San Francisco, CA). The IgG was generated as an effectorless mouse IgG2a containing L234A, L235A, and P329G mutations. The Fab was generated with a human constant domain. IgGs and Fabs were expressed by transient transfection of HEK293 cells (Expi293F, Invitrogen) and purified with affinity chromatography followed by SEC using standard methods (MabSelect SuRe or HiTrap Protein G; GE Healthcare, Piscataway, NJ, USA).

### Generation of COS7 cells expressing NOX2, p22, p47, and p67

Full-length cDNAs encoding human *CYBB* (NOX2), *CYBA* (p22), *NCF1* (p47-phox), and *NCF2* (p67-phox) were inserted into pMSCV-GFP, pMSCV-hCD4, pMSCV-mCherry, and pMSCV-puro (Genentech), respectively, in which the marker genes were expressed bicistronically using an internal ribosome entry site (IRES). For the mutant assays, cDNAs coding the wild type and the indicated mutant human *CYBB* (NOX2) with N-terminal tandem twin-strep twin-FLAG tag were inserted into pMSCV-GFP. The Platinum A cell line (Thermo Fisher Scientific) was transiently transfected with these plasmids individually by FuGene 6 (Promega) to generate conditioned media containing retrovirus. COS7 cells were spin-infected at 750 × *g* for 1 h with retrovirus in the presence of 8 µg/mL polybrene (Sigma-Aldrich), and sorted by fluorescence-activated cell sorting (FACS) using BD influx (BD biosciences) and puromycin resistance (5 µg/mL).

### Generation of NOX2 KO and differentiation of HL60 cell line

An HL60 cell line was transiently transfected with GeneArtCripsrNucleaseVectorOFP.hCybb1 coding gRNA sequence (TCAGAGAGTGCTACTGAATA), Cas9 and Orange Fluorescent Protein (OFP). Transfected cells were sorted by FACS for OFP expression, and expanded. The cultured cells were then further sorted several times for negative expression of NOX2 after staining with anti-NOX2 antibody (5 µg/mL, clone 7D5, LSBio) followed by APC-conjugated F(ab’)_2_-goat anti-mouse IgG (2 µg/mL, 17-4010-82, Thermo Fisher Scientific). All HL60 cell lines were differentiated in the culture media (RPMI media containing 10% FBS, GlutaMAX (Thermo Fisher Scientific), and penicillin/streptomycin(Thermo Fisher Scientific)) supplied with 1.3% DMSO (Sigma-Aldrich) for 4–5 days, prior to ROS expression analysis or FACS analysis.

### Human blood samples

Peripheral blood samples from healthy male and female donors at least 18 years of age were kindly provided by the Samples4Science donor program at Genentech. Donors provided written informed consent and sample collection was approved by the Western Institutional Review Board.

### Human neutrophil isolation

Human neutrophils were isolated by density gradient with histopaque 1119 (Sigma-Aldrich) and histopaque 1077 (Sigma-Aldrich). Briefly, 20 mL of whole blood were layered over hisopaque 1077 over 1119 in 50 mL conical tube. The tube was spun at 700 × *g* at room temperature for 30 min without braking during deceleration. The neutrophils were isolated from the interface between histopaque 1077 and 1119. After washing with Dulbecco’s phosphate-buffered saline buffer (DPBS, Thermo Fisher Scientific), the contaminated red blood cells were lysed by resuspending in 5 mL of 0.2% NaCl for 15 s, after which 5 mL 1.6% NaCl was added to stop the lysis. The cells were further washed with DPBS, and used for the ROS expression assay.

### Surface expression analysis, cell-based affinity assay, and internalization assay

To determine NOX2 expression, COS7 cells expressing NOX2 subunits, wild-type and NOX2-deficient differentiated HL60 cells were evaluated by staining with 7G5 IgG at 5 µg/mL in FACS buffer-1 (PBS with 2% FBS and 0.2% NaN_3_) at room temperature for 1 h. After washing with FACS buffer-1 twice, the cells were resuspended in FACS buffer-1 containing the 2 µg/mL of APC-conjugated F(ab’)_2_-goat anti-mouse IgG (17-4010-82, Thermo Fisher Scientific) and incubated at room temperature for 30 min. After washing with FACS buffer-1 twice, the cells were resuspended in FACS buffer-1 and analyzed by BD FACSCelesta, LSRFortessa, or FACSSymphony (BD Biosciences), and data analyses were performed using FlowJo v.10.8.1 software.

For cell-based affinity assay, 7G5 Fab or 7G5 IgG were labeled with Lightning-Link® Rapid Dylight 550 Antibody Labeling Kit (Novus Biologicals, 323-0005), and for internalization assay, 7G5 Fab was labeled with biotin using Lightning-Link® Rapid Type A Biotin Antibody Labeling Kit (Novus Biologicals, 370-0030) according to the manufacturer’s instructions. HL60 cells were differentiated with 1.3% DMSO in complete RPMI 1640 medium for 4 days. 1  ×  10^5^ cells suspended in FACS buffer-2 (PBS containing 0.5% BSA and 2 mM EDTA) containing 25% of human serum were incubated with various concentrations of Dylight 550 conjugated 7G5 Fab or 7G5 IgG, at 37 °C in the presence of 0.1% NaN_3_ for 4 h (cell-based affinity assay) or with biotin-conjugated 7G5 Fab or unconjugated 7G5 IgG at 37 °C in the presence or absence of 0.1% NaN_3_ for 1.5 h (internalization assay), then washed twice with FACS buffer-2. For incubation with biotin-conjugated 7G5 Fab or unconjugated 7G5 IgG, the cells were further stained with Alexa Fluor 647 Streptavidin (5 µg/mL, Biolegend, 405237) or Alexa Fluor® 647 AffiniPure F(ab’)_2_ Fragment Goat Anti-Mouse IgG (Jackson Immunoresearch Laboratories, 115-606-071, 1:500, for internalization assay) respectively, at 4 °C for 15 min, then washed twice with FACS buffer-2. The cells were resuspended in FACS buffer-2 with propidium iodide (BD Biosciences 556463, 0.5 µg/ml) for analysis on a BD FACSCelesta cell analyzer (BD Biosciences), and data analyses were performed using FlowJo v.10.6.1 software. MFIs for the IgG range from ∼380 to ∼19,000 and MFIs for the Fab range from ∼100 to ∼600. The binding EC50 of each antibody and maximum binding mean fluorescence intensity (MFI) were calculated by fitting the cell binding data using Prism 9 for MacOS software, One Site-Fit Ki model, to determine the affinity of each antibody.

### ROS production analysis

After washing with PBS or DPBS, cells (COS7, HL60 or human neutrophils) were seeded at 0.1 million cells/well in 200 µL of luminol buffer (5 mM glucose, 1 mM MgCl_2_, 0.5 mM CaCl_2_) containing 0.33 mM histidine acetate and 2.5 mM NaCl (supplemented for normalizing the buffer effect of antibody solution), 50 µM isoluminol (for human neutrophils and HL60 cell line, Sigma-Aldrich) or 25% Diogenes (for COS7 cell line, National Diagnostics), in the presence or absence of the indicated concentration of antibodies or apocynin (Abcam). For the NOX2 mutant assay, histidine acetate and NaCl were not supplemented. After incubation at room temperature for 1 h, the cells were stimulated with 100 nM Phorbol 12-myristate 13-acetate (PMA, Sigma-Aldrich), and the luminescence was measured using a GloMAX luminometer (Promega) at room temperature. For the NOX2 mutant assay, the cell number was analyzed by CellTiter-Glo assay (Promega) after the ROS production analyses. The ROS production data was normalized by both the geometric mean fluorescence intensity of NOX2 surface staining and the RLU of CellTiter-Glo assay.

### Cell-free ROS production assay

HL60 cells differentiated for 4–5 days were collected by centrifugation at 5000  ×  *g* for 15 min. The cell pellet was homogenized with a dounce homogenizer and sonicated in PBS at pH 7.4 supplemented with Roche protease inhibitor tablets and 1 µg/mL benzonase. Lysed cells were centrifuged at 500 × *g* for 15 min and the resulting supernatant containing the membrane fractions was further centrifuged at 100,000  ×  *g* for 1 h. Membrane pellets were resuspended in PBS and stored at −80 °C.

The setup of the cell-free assay followed the recommendations outlined in this publication^21^. HL60 membranes of roughly ∼1.3  ×  10^6^ cells/well were supplemented with the cytosolic NOX2 subunits full-length p47, full-length p67, and full-length Rac1 Q61L (all at 1.5 µM), lithium dodecyl sulfate (LiDS, 120 µM), FAD (10 µM), MgCl_2_ (1 mM), 50% Diogenes (National Diagnostics) in the presence or absence of antibody (7G5 IgG/7G5 Fab/anti-gp120 IgG at 1 µM) as indicated in Supplementary Fig. 9i. The reaction was initiated by the addition of 250 µM NADPH, and the luminescence was measured using a GloMAX luminometer (Promega) at room temperature. Analysis was performed in Prism 9, and the unpaired two-sided Student’s *t* test was performed to compare samples in the presence of antibody to the control with no antibodies added.

### Reporting summary

Further information on research design is available in the Nature Research Reporting Summary linked to this article.

## Supplementary information

Supplementary Information

Reporting Summary

## Data Availability

All data included in the paper and the supplementary information files are available. Sequence information of NOX2 and p22 was obtained from the Uniprot database (NOX2: P04839, p22: Q86SL0). The 3D cryo-EM map has been deposited into the Electron Microscopy Data Bank under accession code EMD-26383. The coordinates of NOX2 core-7G5 complex have been deposited in the PDB with accession code 7U8G. Source data are provided with this paper.

## Source data

Source Data
