# Supplementary material for: Structure of the core human NADPH oxidase NOX2
Source: Nat Commun. 2022 Oct 14;13:6079. doi: 10.1038/s41467-022-33711-0 (PMC9568551; doi:10.1038/s41467-022-33711-0)

Supplementary Information

Structure of the core human phagocyte NADPH oxidase NOX2

Supplementary Figures

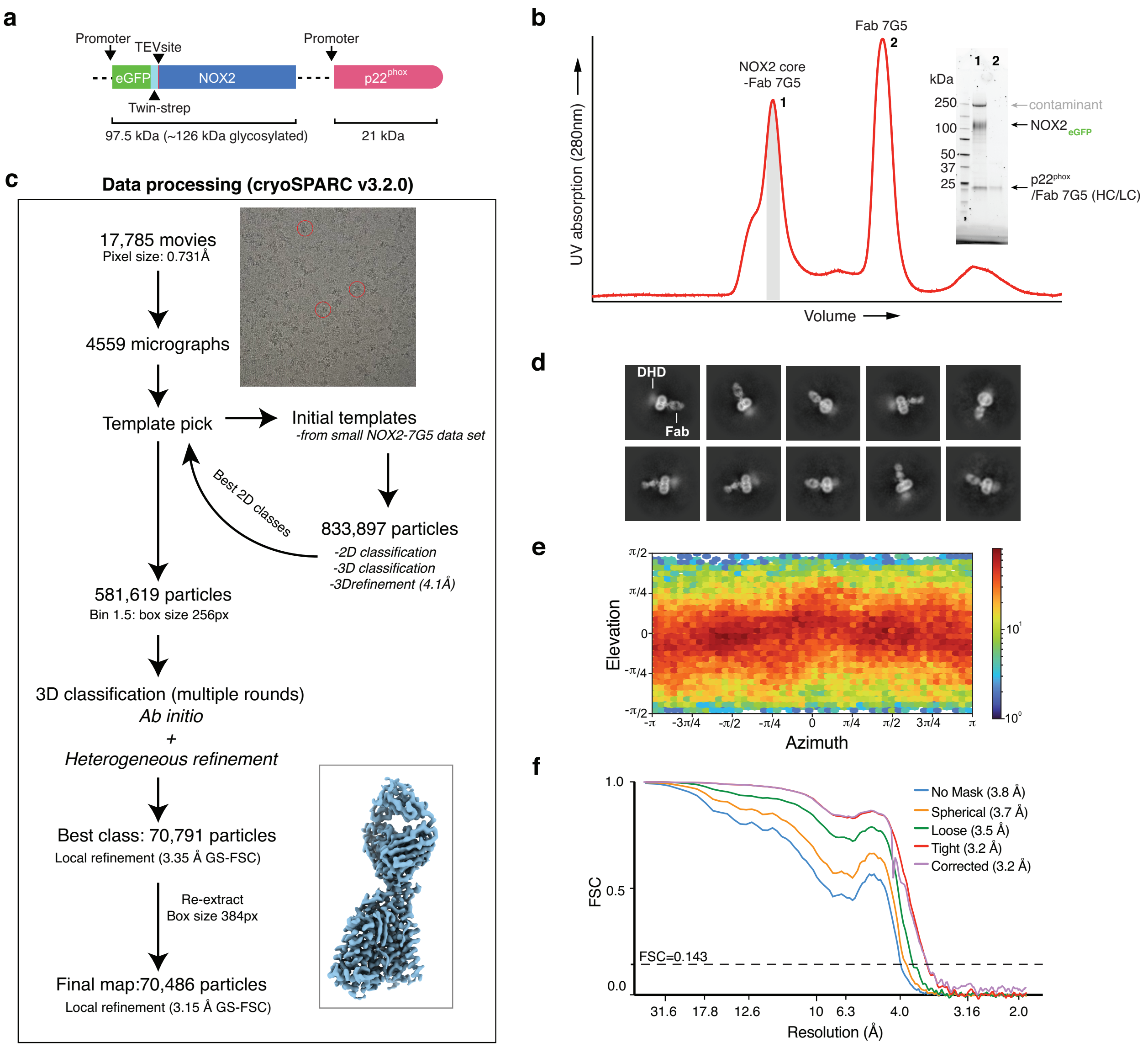

Supplementary Figure 1. Biochemistry and cryo-EM data processing of the NOX2 core complex. a. Schematic illustration of the NOX2 core bicistronic construct. **b.** Size-exclusion chromatogram of the purified NOX2 core-Fab 7G5 complex with representative SDS-PAGE of the peak 1 fraction that was used for structure determination (lane 1, highlighted in grey on the chromatogram) and the second peak where excess Fab 7G5 elutes (lane 2). The ~250 kDa

contaminant was identified as Acetyl-CoA carboxylase 1, which did not form a complex with NOX2 core-7G5. **c.** Cryo-EM data processing workflow where cryoSPARC v3.2.0 was used for particle picking, 2D classification and multiple rounds of 3D classification to reach a final three-dimensional reconstruction of NOX2 core-Fab 7G5 at 3.2 Å. **d.** Representative 2D class averages of NOX2 core-Fab 7G5 complex. **e.** Angular particle distribution of projections. **f.** Gold-standard Fourier shell correlation (GS-FSC).

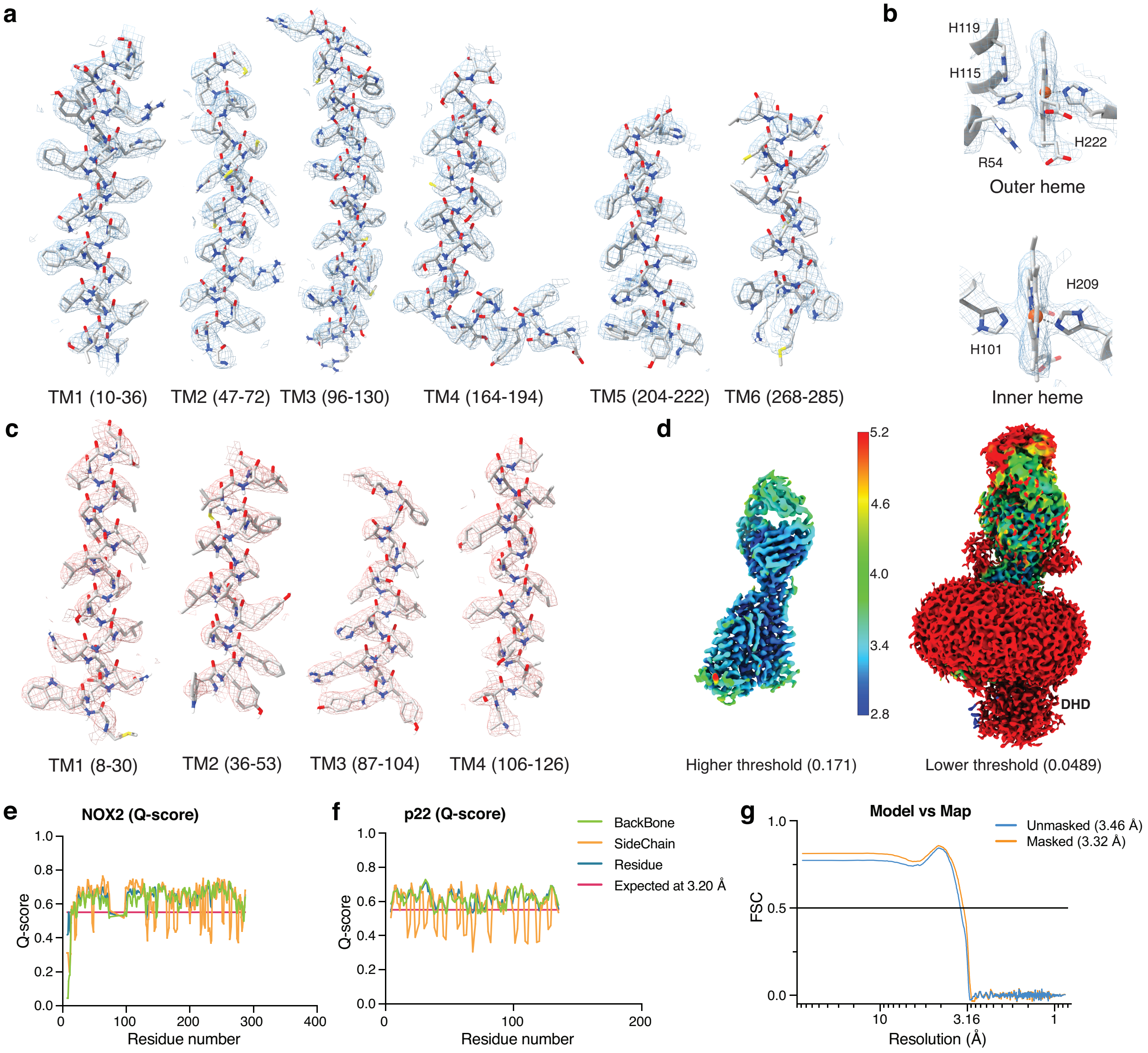

Supplementary Figure 2. NOX2 model vs map validation. a. NOX2 coordinates of each transmembrane helix (TM1-6) are colored in blue mesh. **b.** The two heme molecules that are coordinated by histidines and residues of the oxygen reduction center, all located in NOX2 are colored in blue mesh. **c.** p22^{phox} coordinates of each transmembrane helix (TM1-4) are colored in red mesh. **d.** Local resolution estimate. **e, f.** Q-score plot of NOX2 **(e)** and p22 **(f)**. **g.** Fourier shell correlation plot of model vs map

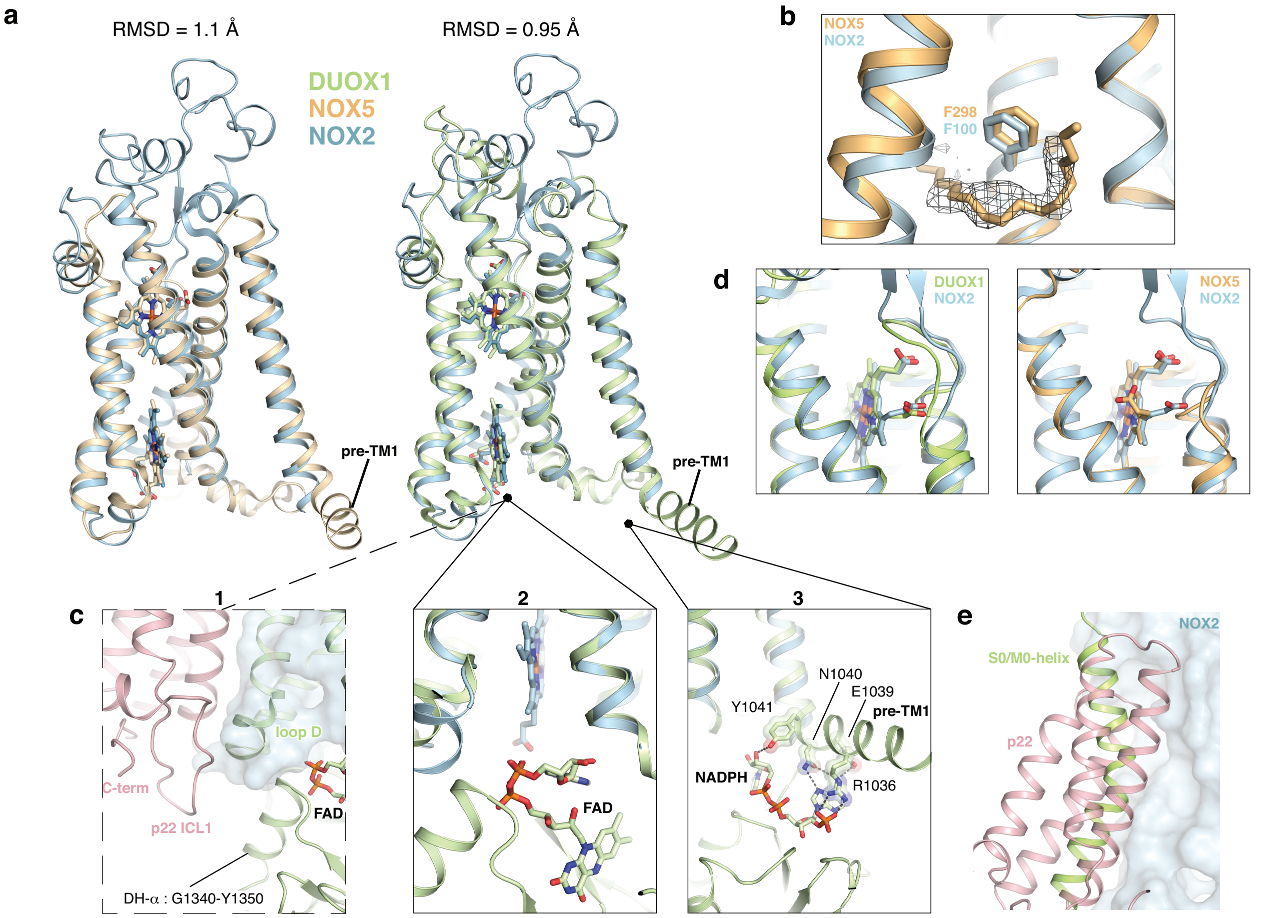

Supplementary Figure 3. Comparison of NOX2 structure to *cs*NOX5 and DUOX1 structures. **a.** Superposition of the NOX2 TMD of NOX2 (blue), human DUOX1 (green, PDB 7d3f) and *cs*NOX5 (light orange, PDB:5o0t), with RMSD values of 1.1 Å for *cs*NOX5, 0.97 Å for mouse DUOX1 and 0.95 Å for DUOX1. The pre-TM1 helix seen in csNOX5 and DUOX1 is not observed in NOX2, and a putative NOX2 pre-TM1 helix would only extend by 7 residues, as highlighted in **supplementary figure 5**. **b.** An alkyl chain modeled in csNOX5 (light orange, PDB:5o0t) fits within the NOX2 cryo-EM density (grey mesh) of superposed NOX2 (blue), supporting prior structural observations of this interface harboring a lipid pocket. The sidechain of Phe100, conserved in all NOX/DUOX members is shown in close proximity of the modeled alkyl chain. **c.** Overlay of the NOX2 core and DUOX1 (green, PDB: 7d3f), places the DHD underneath NOX2 and FAD close to the inner heme. Panel 1 shows that p22 ICL1 is in close proximity of DH-α helix of DUOX1.

The equivalent DH-α region in NOX2 been shown to bind to the cytosolic subunit p67. Panel 2 includes close-up view with FAD closely located to the inner heme. Panel 3 includes a close-up view of NADPH where side chains from pre-TM1 helix of DUOX, not conserved in NOX2, forms hydrogen bonds with NADPH. **d.** Comparison of outer heme - ECL interactions and capping in NOX2 with DUOX1 (left panel, PDB 7d3f) and csNOX5 (right panel, PDB 5o0t) **e.** Overlay of DUOX1 (green) with the NOX2 core, showing that the M0/S0-helix is located at the same interface as p22.

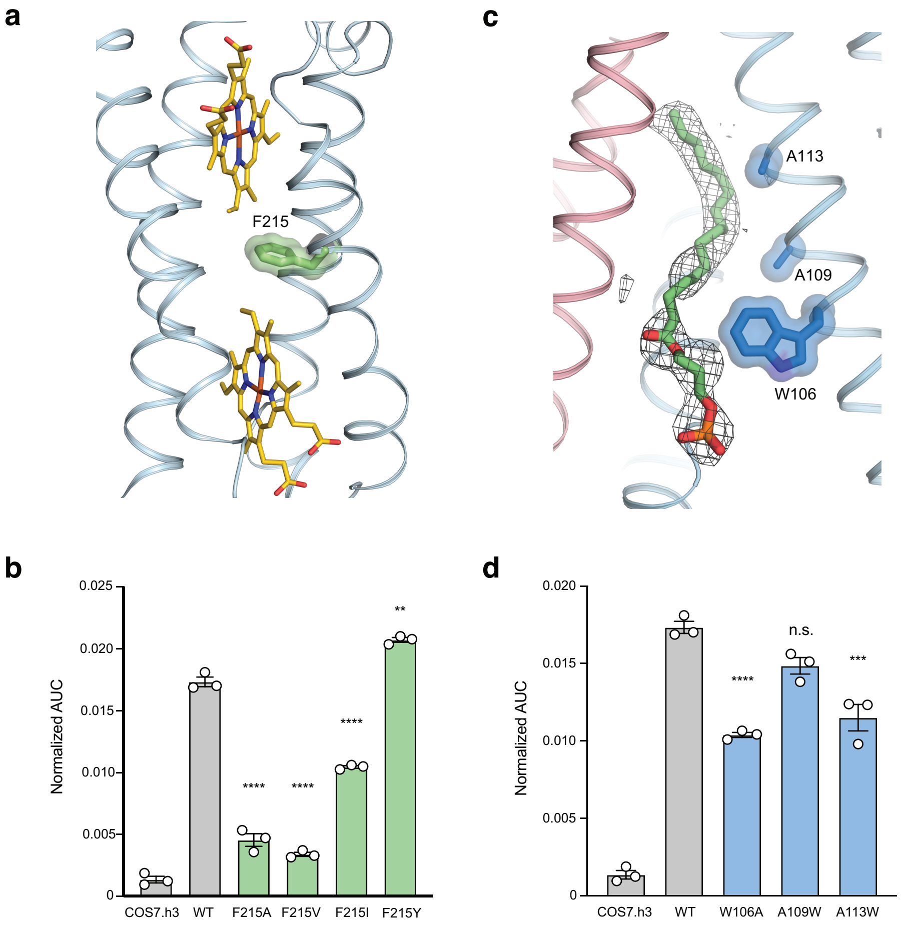

Supplementary Figure 4. Functional assessment of NOX2 F215, W106, A109 and A113 mutations. a. Phe215 is located between the inner heme and the outer heme and is potentially involved in electron transfer. **b.** Point mutations Phe215Ala, Phe215Val and Phe215Ile lead to strongly reduced NOX2 ROS production, while mutation Phe215Tyr slightly increases ROS production. The bar graph represents the area under the curve (AUC) of ROS production as measured by relative light units (RLU) in PMA-stimulated COS7 cells expressed with p22/p47/p67 (COS7.h3) or NOX2/p22/p47/p67 (WT with or without NOX2 point mutations), as detailed in the methods section (n=3 biologically independent samples, mean ± SD). Data has been normalized by geometric mean fluorescence intensity of NOX2 surface staining and RLU of CellTiterGlo assay. **c.** Residues Trp106, Ala109 and Ala113 are located at the interface between NOX2 and

p22 and complement the shape of a lipid wedged in between p22 and NOX2. **d.** Mutation Trp106Ala and Ala113Trp reduce NOX2 ROS production by ~40% and 30%, respectively, while mutation Ala109Trp does not affect ROS production. Bar graphs were generated as described for **(b)**. Each mutant with reported p-value was generated from comparison to WT (second bar from the left) by unpaired two-sided t-test, n.s. = no statistical significance, $^{**}P < 0.01$, $^{***}P < 0.001$, $^{****}P < 0.0001$. Source data are available in the Source Data File.

```
                                                        pre-TM1            TM1
NOX2   ---------- ---------- ---------- ------MGNW AVNEGLSIFV ILVWLGLNVF   24
NOX1   ---------- ---------- ---------- ------MGNW VVNHWFSVLF LVVWLGLNVF   24
NOX3   ---------- ---------- ---------- -----MMGCW ILNEGLSTIL VLSWLGINFY   25
NOX4   ---------- ---------- ---------- --MAVSWRSW LANEGVKHLC LFIWLSMNVL   28
NOX5   ---------- ---------- ---------RP RRPRQLTRAY WHNHRSQLFC LATYAGLHVL  251
DUOX1  IRRRFGKKVT SFQPLLFTEA HREKFQRSCL HQTVQQFKRF IENYRRHIGC VAVFYAIAGG 1057
DUOX2  LKKRFGKKAA VPTPRLYTEA LQEKMQRGFL AQKLQQYKRF VENYRRHIVC VAIFSAICVG 1054

                  loop A            TM2                         loop B
NOX2   LFVWYYRVYD IPPKFFYTRK LLGSALALAR APAACLNFNC MLILLPVCRN LLSFLRGSSA   84
NOX1   LFVDAFLKYE KADKYYYTRK ILGSTLACAR ASALCLNFNS TLILLPVCRN LLSFLRGTCS   84
NOX3   LFIDTFYWYE EEESFHYTRV ILGSTLAWAR ASALCLNFNC MLILIPVSRN LISFIRGTSI   85
NOX4   LFWKTFLLYN QGPEYHYLHQ MLGLGLCLSR ASASVLNLNC SLILLPMCRT LLAYLRGSQK   88
NOX5   LFGLAA---- -------SAHR DLGASVMVAK GCGQCLNFDC SFIAVLMLRR CLTWLRATWL  301
DUOX1  LFLERAYYYA FAAHHTGITD TTRVGIILSR GTAASISFMF SYILLTMCRN LITFLRETFL 1117
DUOX2  VFADRAYYYG FASPPSDIAQ TTLVGIILSR GTAASVSFMF SYILLTMCRN LITFLRETFL 1114

                             TM3                               loop C -α1
NOX2   CCSTRVRRQL DRNLTFHKMV AWMIALHSAI HTIAHLFNVE WCVNARVNNS DPYSVALSEL  144
NOX1   FCSRTLRKQL DHNLTFHKLV AYMICLHTAI HIIAHLFNFD CYSRSRQATD GSLASILSSL  144
NOX3   CCRGPWRRQL DKNLRFHKLV AYGIAVNATI HIVAHFFNLE RYHWSQSEEA QGLLAALSKL  145
NOX4   VPSRRTRRLL DKSRTFHITC GVTICIFSGV HVAAHLVNAL NFSVNYSEDF ----------  138
NOX5   AQV----LPL DQNIQFHQLM GYVVVGLSLV HTVAHTVNFV LQAQAEASPF QFWELLLT--  355
DUOX1  NRY----VPF DAAVDFHRLI ASTAIVLTVL HSVGHVVNVY LFSISPLSVL SCLF---PGL 1170
DUOX2  NRY----VPF DAAVDFHRWI AMAAVVLAIL HSAGHAVNVY IFSVSPLSLL ACIF---PNV 1167

            loop C         loop C -α2               TM4             loop D
NOX2   GD--RQNESY LNFARKRIKN PEGGLYLAVT LLAGITGVVI TLCLILIITS STKTIRR-SY  201
NOX1   SHDEKKGGSW LNPIQSRNT- --TVEYVTFT SIAGLTGVIM TIALILMVTS ATEFIRR-SY  200
NOX3   GN--TPNESY LNPVRTFPTN TTTE---LLR TIAGVTGLVI SLALVLIMTS STEFIRQ-AS  199
NOX4   ---------VE LNAARYRDED P---RKLLFT TVPGLTGVCM VVVLFLMITA STYAIRV-SN  186
NOX5   ---TRPGI-- ---------- ----G--WVH GSASPTGVAL LLLLLLMFIC SSSCIRRSGH  394
DUOX1  ---------F H---DDGSEL PQKYYWWFFQ TVPGLTGVVL LLILAIMYVF ASHHFRR-RS 1217
DUOX2  ---------F V---NDGSKL PQKFYWWFFQ TVPGMTGVLL LLVLAIMYVF ASHHFRR-RS 1214

                  TM5                  loop E -β1          loop E
NOX2   FEVFWYTHHL FVIFFIGLAI HGAERIVRGQ TAESLAV-HN ITVC---EQK ISEWGKIKE-  247
NOX1   FEVFWYTHHL FIFYILGLGI HGIGGIVRGQ TEESMNESHP RK-C---AES FEMWDDRDSH  246
NOX3   YELFWYTHHV FIVFFLSLAI HGTGRIVRGQ TQDSLSL-HN ITFC---RDR YAEWQTVAQ-  245
NOX4   YDIFWYTHNL FFVFYMLLTL HVSGGLLKYQ TNLDT---HP PG-CISLNRT SSQNISLPEY  242
NOX5   FEVFVYWTHLS YLLVWLLLIF HG-------- ---------- ---------- ----------  416
DUOX1  FRGFWLTHHL YILLYVLLII HGSFALIQL- ---------- ---------- ---------- 1246
DUOX2  FRGFWLTHHL YILLYALLII HGSYALIQL- ---------- ---------- ---------- 1243

                                              loop E -β2         TM6
NOX2   ---------- ---------- ---------CP I-PQFAGNPP MTWKWIVGPM FLYLCERLVR  287
NOX1   ---------- ---------- ---------CR R-PKFEGHPP ESWKWILAPV ILYICERILR  287
NOX3   ---------- ---------- ---------CP V-PQFSGKEP SAWKWILGPV VLYACERIIR  285
NOX4   FSEHFHEPFP EGFSKPAEFT QHKFVKI-CM EEPRFQANFP QTWLWISGPL CLYCAERLYR  301
NOX5   ---------- ---------- ---------- ---------P NFWKWLLVPG ILFFLEKAIG  437
DUOX1  ---------- ---------- ---------- ---------P RFHIFFLVPA IIYGGDKLVS 1267
DUOX2  ---------- ---------- ---------- ---------P TFHIYFLVPA IIYGGDKLVS 1264
```

DH domain

```
NOX2  FWRSQ-QKVV ITKVVTHPFK TIELQMK-KK GFKMEVGQYI FVKCPKVSKL EWHPFTLTSA  345
NOX1  FYRSQ-QKVV ITKVVMHPSK VLELQMN-KR GFSMEVGQYI FVNCPSISLL EWHPFTLTSA  345
NOX3  FWRFQ-QEVV ITKVVSHPSG VLELHMK-KR GFKMAPGQYI LVQCPAISSL EWHPFTLTSA  343
NOX4  YIRSN-KPVT IISVMSHPSD VMEIRMV-KE NFKARPGQYI TLHCPSVSAL ENHPFTLTMC  359
NOX5  LAVSRMAAVC IMEVNLLPSK VTHLLIKRPP FFHYRPGDYL YLNIPTIARY EWHPFTISSA  497
DUOX1 LSRKK-VEIS VVKAELLPSG VTHLRFQRPQ GFEYKSGQWV RIACLALGTT EYHPFTLTSA 1326
DUOX2 LSRKK-VEIS VVKAELLPSG VTYLQFQRPQ GFEYKSGQWV RIACLALGTT EYHPFTLTSA 1323

NOX2  PEE--DFFSI HIRIVGDWTE GLFNACGC-- ---------- ---------- -----DKQEF  376
NOX1  PEE--DFFSI HIRAAGDWTE NLIRAFEQ-- ---------- ---------- -----Q----  372
NOX3  PQE--DFFSV HIRAAGDWTA ALLEAFGA-- ---------- ---------- -----EGQAL  374
NOX4  PTETKATFGV HLKIVGDWTE RFRDLLLPPS SQD------- ---------- ----SEILPF  398
NOX5  PEQ-KDTIWL HIRSQGQWTN RLYESFKASD PLGRGSKRLS RSVTMRKSQR SSKGSEILLE  556
DUOX1 PHE--DTLSL HIRAAGPWTT RLREIYSAPT GDR------- ---------- ---------- 1357
DUOX2 PHE--DTLSL HIRAVGPWTT RLREIYSSPK GNG------- ---------- ---------- 1354

NOX2  QDAWKLPKIA VDGPFGTASE DVFSYEVVML VGAGIGVTPF ASILKSVWYK YCNN------  430
NOX1  --YSPIPRIE VDGPFGTASE DVFQYEVAVL VGAGIGVTPF ASILKSIWYK FQCA------  424
NOX3  QEPWSLPRLA VDGPFGTALT DVFHYPVCVC VAAGIGVTPF AALLKSIWYK CSEA------  428
NOX4  IQSRNYPKLY IDGPFGSPFE ESLNYEVSLC VAGGIGVTPF ASILNTLLD- ---D------  448
NOX5  KHKFCNIKCY IDGPYGTPTR RIFASEHAVL IGAGIGITPF ASILQSIMYR HQKRKHTCPS  616
DUOX1 --CARYPKLY LDGPFGEGHQ EWHKFEVSVL VGGGIGVTPF ASILKDLVFK SSVS------ 1409
DUOX2 --CAGYPKLY LDGPFGEGHQ EWHKFEVSVL VGGGIGVTPF ASILKDLVFK SSLG------ 1406

NOX2  ---------A TNLKLKKIYF YWLCRDTHAF EWFADLLQLL ESQMQ-ERNN AGFLSYNIYL  480
NOX1  ---------D HNLKTKKIYF YWICRETGAF SWFNNLLTSL EQEME-ELGK VGFLNYRLFL  474
NOX3  ---------Q TPLKLSKVYF YWICRDARAF EWFADLLLSL ETRMS-EQGK THFLSYHIFL  478
NOX4  ---------W KPYKLRRLYF IWVCRDIQSF RWFADLLCML HNKFW-QENR PDYVNIQLYL  498
NOX5  CQHSWIEGVQ DNMKLHKVDF IWINRDQRSF EWFVSLLTKL EMDQAEEAQY GRFLELHMYM  676
DUOX1 ---------C -QVFCKKIYF IWVTRTQRQF EWLADIIREV ----E-ENDH QDLVSVHIYI 1454
DUOX2 ---------S -QMLCKKIYF IWVTRTQRQF EWLADIIQEV ----E-ENDH QDLVSVHIYV 1451

NOX2  TGWDESQANH FAV------H H--DEEKDVI TGLKQKTLYG RPNWDNEFKT IASQHPN-TR  531
NOX1  TGWDSNIVGH AAL------N F--DKATDIV TGLKQKTSFG RPMWDNEFST IATSHPK-SV  525
NOX3  TGWDENQALH IAL------H W--DENTDVI TGLKQKTFYG RPNWNNEFKQ IAYNHPS-SS  529
NOX4  SQTDGIQKII ---------- ------GEKY HALNSRLFIG RPRWKLLFDE IAKYNRG-KT  541
NOX5  TSALGKNDMK AIGLQMALDL LANKEKKDSI TGLQTRTQPG RPDWSKVFQK VAAEKKG--K  734
DUOX1 TQLAEKFDLR TTMLYICERH FQKVLNRSLF TGLRSITHFG RPPFEPFFNS LQEVHPQVRK 1514
DUOX2 TQLAEKFDLR TTMLYICERH FQKVLNRSLF TGLRSITHFG RPPFEPFFNS LQEVHPQVRK 1511

NOX2  IGVFLCGPEA LAETLSKQSI SNSESGPRGV HFIFNKENF-                        570
NOX1  VGVFLCGPRT LAKSLRKCCH RYSSLDPRKV QFYFNKENF-                        564
NOX3  IGVFFCGPKA LSRTLQKMCH LYSSADPRGV HFYYNKESF-                        568
NOX4  VGVFCCGPNS LSKTLHKLSN QNNS---YGT RFEYNKESFS                        578
NOX5  VQVFFCGSPA LAKVLKGHCE KF-------- GFRFFQENF-                        765
DUOX1 IGVFSCGPPG MTKNVEKACQ LINRQD--RT HFSHHYENF-                       1551
DUOX2 IGVFSCGPPG MTKNVEKACQ LVNRQD--RA HFMHHYENF-                       1548
```

Supplementary Figure 5. Sequence alignment of NOX2 with other members of the NOX superfamily. Sequence alignment of NOX2 (uniprot: P04839) with NOX1/3/4/5 (uniprot:

Q9Y5S8, Q9HBY0, Q9NPH5, Q96PH1, respectively) and DUOX1/2 (uniprot: Q9NRD9, Q9NRD8). Sequences of NOX1/3/4 are full-length, while part of the NOX5 and DUOX1/2 sequences prior to TM1 have been omitted. The sequences were aligned with Clustal Omega and manually adjusted. The secondary structure allocation is based on NOX2 structure, with the structured pre-TM1 region observed in NOX5 and DUOX1 highlighted as a gray helix. Coloring or shading is as follows: histidines that coordinate the heme molecules are colored white in blue boxes, while residues at the oxygen reduction center are colored black in blue boxes. Glycosylation sites are colored purple and the two cysteines participating in a disulfide bond are in red boxes. Residues in the ECLs that are conserved in NOX1-4 that participate in a network of polar interactions connecting the outer heme and ECLs are colored in light orange boxes.

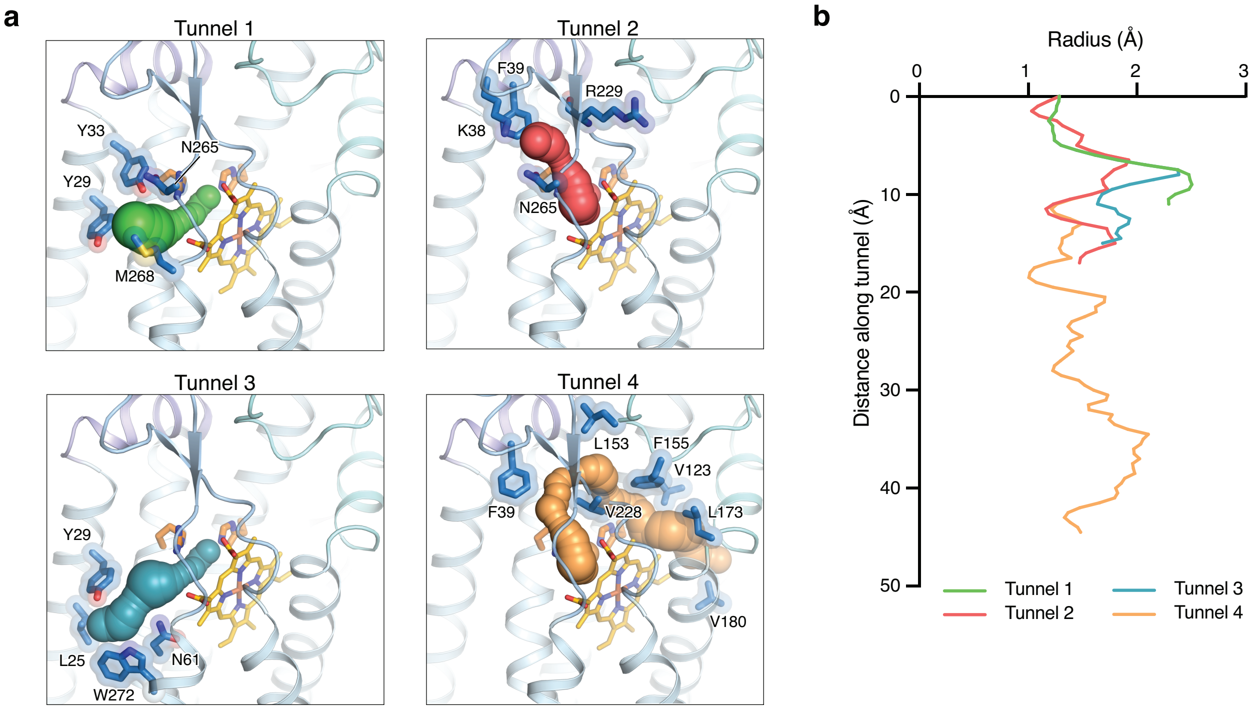

Supplementary Figure 6. CAVER analysis reveals four tunnels that start from the reduction center. a. Starting from His115, tunnels 1, 2, 3 and 4 are shown in green, red, blue and orange, respectively. Residues lining the tunnels are shown as blue sticks in transparent surface. **b.** Radius of each tunnel as tunnel distance from His115 increases.

a

Protein	PDB-chain	Z-score	RMSD
Copper storage protein (CSP3)	5fig-F	9.4	2.2
Tweety homolog 2 (TTYH2)	7p54-B	8.0	2.9
Claudin-9	6ov2-A	7.9	3.5
AMPAR regulatory protein (TARP)	7ryz-A	7.7	3.3
$Ca_v\gamma1$ auxiliary subunit	7jpw-E	7.2	3.2

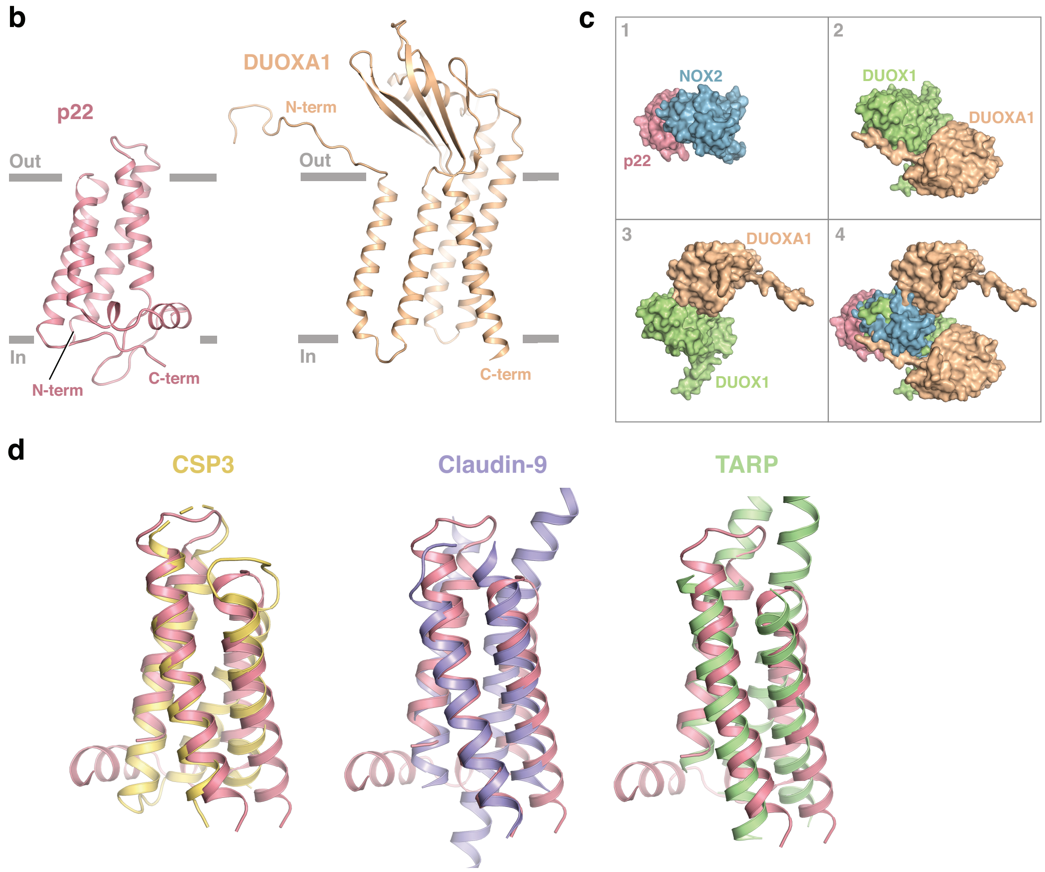

Supplementary Figure 7. Structural homologs of p22 and comparison of p22 to DUOXA1. **a.** Table of top hits from DALI search. **b.** Comparison of the fold and structure of auxiliary proteins p22 and DUOXA1. **c.** The interface between NOX2-p22 (panel 1) is different compared to the two interfaces of DUOX1-DUOXA1 (panel 2 and 3) as seen when superposing NOX2 and DUOX1 (panel 4). NOX2 was superposed with DUOX1 (PDB: 7d3f), which is a dimer of dimers (DUOX1-DUOXA1- DUOX1-DUOXA1). Only one DUOX1 is illustrated in panel 2-4 to simplify the

illustration of the two interfaces that DUOXA1 binds to. **d.** Superposition of three structural homologs of p22, all of which have diverse functions. Copper storage protein 3 (CSP3, PDB: 5fig-F, yellow) exists as a tetramer and is the closest structural homolog. Claudin-9 (PDB:6ov2-A, light blue) is part of the claudin superfamily that play a role in lateral adhesion and ion permeation between cells. Transmembrane AMPAR regulatory protein (TARP, PDB:7ryz-A, green) is an important regulator of AMPA receptors.

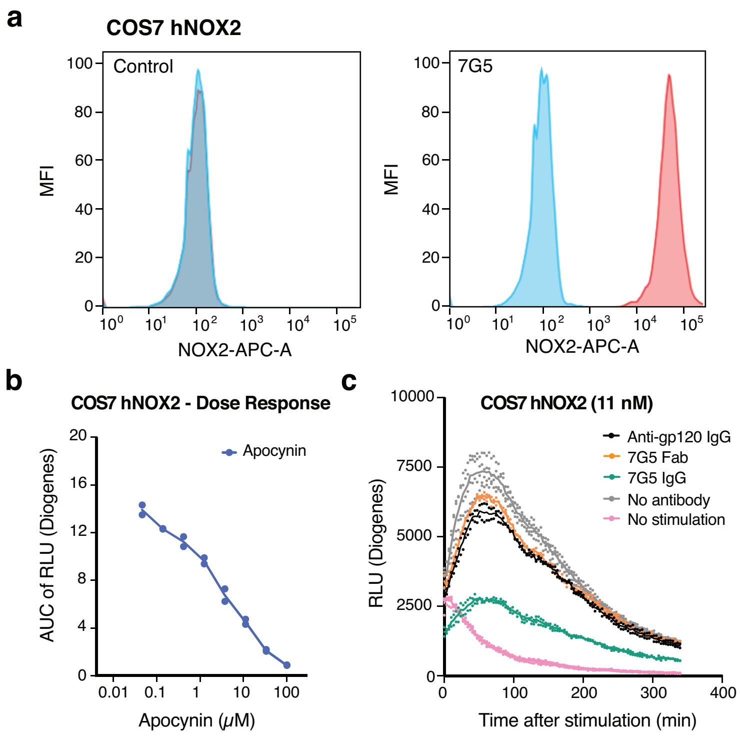

Supplementary Figure 8. Characterization of anti-NOX2 7G5 in COS7 cells. a. 7G5 IgG binds to recombinant hNOX2 expressed in COS7 cells. COS7 cells expressing partial hNOX2 (p22/p47/p67, left panel) or complete hNOX2 (NOX2-p22-p47-p67, right panel) are either stained with 7G5 IgG (red) or isotype control antibody (blue). An APC-conjugated secondary antibody was used to detect 7G5 IgG. **b**. Apocynin inhibits extracellular ROS that is produced by COS7 cells expressing recombinant hNOX2. Each replicate is the area under the curve (AUC) of extracellular ROS measured by a ROS production assay at a given concentration of apocynin (x-axis). The amount of ROS in the ROS production assay is represented as relative light units (RLU). Data represent two replicates per condition where mean values are represented as a solid line. **c**. Extracellular ROS production over time in the presence of 11 nM 7G5 IgG or Fab in COS7 cells. The amount of ROS is measured in relative light units (RLU) in COS7 cells expressing recombinant NOX2 enzymatic complex (NOX2-p22-p47-p67). Data represent two replicates per

time point where mean values are represented as a solid line. Source data are available in the Source Data File.

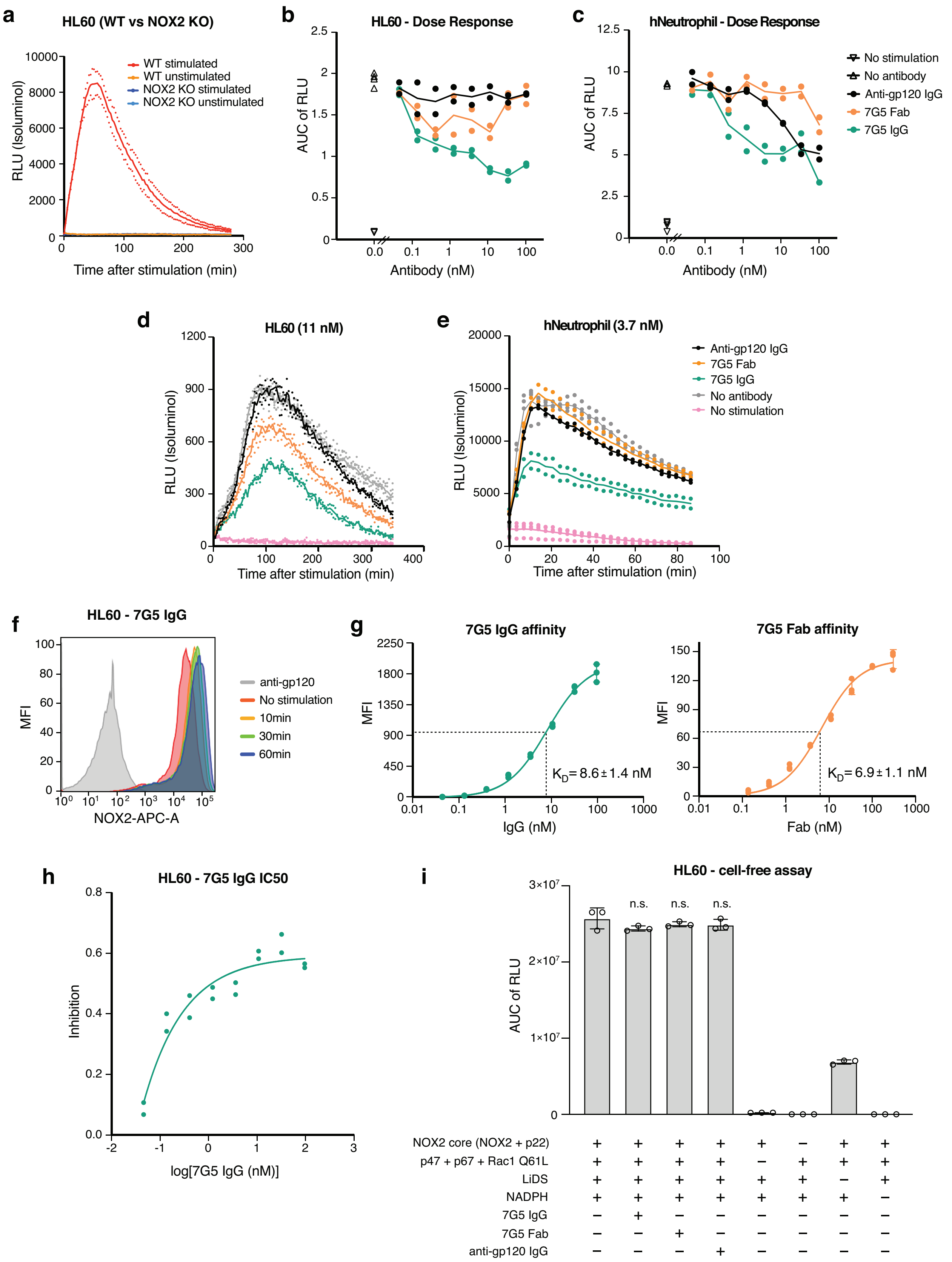

a
HL60 (WT vs NOX2 KO)
WT stimulated
WT unstimulated
NOX2 KO stimulated
NOX2 KO unstimulated
RLU (Isoluminol)
Time after stimulation (min)
b
HL60 - Dose Response
AUC of RLU
Antibody (nM)
c
hNeutrophil - Dose Response
No stimulation
No antibody
Anti-gp120 IgG
7G5 Fab
7G5 IgG
AUC of RLU
Antibody (nM)
d
HL60 (11 nM)
RLU (Isoluminol)
Time after stimulation (min)
e
hNeutrophil (3.7 nM)
Anti-gp120 IgG
7G5 Fab
7G5 IgG
No antibody
No stimulation
RLU (Isoluminol)
Time after stimulation (min)
f
HL60 - 7G5 IgG
MFI
NOX2-APC-A
anti-gp120
No stimulation
10min
30min
60min
g
7G5 IgG affinity
MFI
K$_D$= 8.6 ± 1.4 nM
IgG (nM)
7G5 Fab affinity
MFI
K$_D$= 6.9 ± 1.1 nM
Fab (nM)
h
HL60 - 7G5 IgG IC50
Inhibition
log[7G5 IgG (nM)]
i
HL60 - cell-free assay
AUC of RLU
n.s.
n.s.
n.s.
NOX2 core (NOX2 + p22) + + + + + – + +
p47 + p67 + Rac1 Q61L + + + + – + + +
LiDS + + + + + + – +
NADPH + + + + + + + –
7G5 IgG – + – – – – – –
7G5 Fab – – + – – – – –
anti-gp120 IgG – – – + – – – –

Supplementary Figure 9. Characterization of anti-NOX2 7G5 in HL60 cells and human neutrophils. a. Extracellular ROS production in wild type and NOX2-deficient HL60 cells (NOX2 KO HL60 cells). The ROS production is close to absent in PMA-stimulated NOX2 KO HL60 cells, revealing that ROS production in HL60 cells results primarily from NOX2 activity. Data represent two replicates per time point with mean value plotted as a solid line. The amount of ROS is measured as relative light units (RLU). **b-c** Extracellular ROS production assay of HL60 cells **(b)** and human neutrophils **(c)**. Inhibition is observed when the concentration of 7G5 IgG (green), but not 7G5 Fab (orange), increases. Data represent two replicates per condition with solid line representing the mean value. ROS production in untreated cells without and with PMA stimulation is shown by downward (four replicates) and upward (four replicates) triangles, respectively. Each replicate is the area under the curve (AUC) of RLU from ROS production assay as illustrated in **(d, e). d-e.** Extracellular ROS over time in the presence of 11nM 7G5 IgG or Fab in HL60 cells **(d)** or 3.7nM 7G5 IgG or Fab in human neutrophils **(e)**. Data represent two replicates per time point with mean value plotted as a solid line, similar to **(a)**. **f.** Cell surface staining of HL60 cells with 7G5 IgG before (red) and after PMA stimulation (10min – orange, 30min – green, 60min – blue). The 7G5 IgG shows similar cell surface binding to inactive and active NOX2. **g.** Cell-based affinity of 7G5 IgG (left) and 7G5 Fab (right), as measured by FACS using HL60 in the presence of sodium azide, an internalization inhibitor. Data represent mean fluorescent intensity (MFI) with three replicates per condition. The curve (solid line) is fitted to the mean value, with error bars representing standard deviation (S.D). **h**. A one site Ki fit of the 7G5 IgG data shown in **(b)** was used to estimate the approximate half-maximal concentration of 7G5 IgG-mediated ROS inhibition. Data represent two replicates per condition with curve (solid line) fitted to the mean value. **i.** Cell-free ROS production assay of native NOX2 in HL60 cell membranes. ROS production from NOX2 in HL60 membranes is observed when NOX2 is supplemented with LiDS and the three cytosolic subunits: p47, p67 and RacQ61L. Addition of 7G5 IgG ($p=0.7471$), 7G5

Fab ($p=0.8445$) and anti-gp120 IgG ($p=0.8312$) does not inhibit ROS production in a cell-free assay. Each condition of NOX2 enzymatic complex either with 7G5 IgG, 7G5 Fab or anti-gp120 IgG that has reported p-values was generated from comparison to NOX2 enzymatic complex (first bar on the bar graph starting from the left). The amount of ROS is measured in RLU and each replicate represents the AUC of RLU (n=3 biologically independent samples, mean ± SD). Each condition of NOX2 in the presence of 7G5 IgG, 7G5 Fab or anti-gp120 IgG was generated from comparison to NOX2 only (first bar from the left) by unpaired two-sided t-test, n.s. = no statistical significance (statistical significance cutoff $p < 0.05$). Source data are available in the Source Data File.

Supplementary Tables

Supplementary Table 1. Cryo-EM data collection, refinement and validation statistics

	NOX2-Fab7G5
**Data collection and processing**	
Magnification	165,000
Voltage (kV)	300
Electron exposure (e^-/Å^2)	40.4
Defocus range (μm)	0.8 – 1.8
Pixel Size (Å)	0.731
Symmetry imposed	C1
Initial particle images (no.)	581,619
Final particle images (no.)	70,486
Map resolution (Å)	3.15
FSC threshold	0.143
Map resolution range (Å)	2.8 – 31.6
**Refinement**	
Initial model gp91^{phox} (AlphaFold2)	Uniprot P04839
Initial model p22^{phox} (AlphaFold2)	Uniprot P13498
Model resolution (Å)	3.32
FSC threshold	0.5
Model resolution range (Å)	3.0 – 27.9
Map sharpening B factor (Å^2)	-80.0
Model composition	
Non-hydrogen atoms	10,109
Protein residues	621
Ligands	HEM (2), NAG (3), POV (3)
R.m.s deviations	
Bond lengths (Å)	0.003
Bond angles (°)	0.585
Validation	
MolProbity Score	1.39
Clashscore	5.55
Poor rotamers (%)	0.00
CaBLAM outliers (%)	0.83
Ramachandran plot	
Favored	97.55
Allowed	2.45
Disallowed	0.00

Supplementary table 1. Statistics of data collection, three-dimensional reconstruction, and model refinement.

Supplementary table 2. Reported CGD missense mutations in NOX2 and p22

NOX2 subunit	Mutation	Structure Location
p22	Ala16Pro	TM1 (interface)
p22	Gly24Arg/Glu	TM1 (interface)
p22	Gly25Val/Asp	TM1
p22	Gly46Ser	TM2
p22	Leu51Pro/Arg	TM2 (facing a1-helix)
p22	Leu52Pro	TM2 (core)
p22	Glu53Val/Gln	TM2 (core)
p22	Pro55Arg	ICL1
p22	Lys78Asn	a1-helix
p22	Arg90Trp/Gly/Gln/Pro	TM3 (core)
p22	His94Arg	TM3 (core)
p22	Leu96Pro	TM3
p22	Leu105Arg	ECL2 (interface)
p22	Ala117Glu	TM4 (core)
p22	Ser118Arg/Asn	TM4 (core)
p22	Tyr121His	TM4 (core)
p22	Ala124Ser/Val	TM4 (core)
p22	Ala125Thr	TM4 (core)
p22	Glu129Lys	TM4 (core)
p22	Arg139Gln	C-terminal*
p22	Pro156Gln	P-domain*
NOX2	Gly2Trp	N-terminus*
NOX2	Gly9Arg	TM1

NOX2	Trp18Cys	TM1
NOX2	Leu19Val	TM1
NOX2	Gly20Arg	TM1
NOX2	Asn22Ile	TM1
NOX2	Tyr41Asp	Loop A
NOX2	Thr42Arg/Lys	Loop A
NOX2	Leu45Arg	Loop A
NOX2	Leu52Arg	TM2
NOX2	Ala53Asp	TM2
NOX2	Arg54Gly/Met/Ser	TM2
NOX2	Ala55Asp	TM2
NOX2	Pro56Leu	TM2
NOX2	Ala57Glu	TM2
NOX2	Cys59Arg/Phe/Tyr/Trp	TM2
NOX2	Asn63Lys	TM2
NOX2	Cys64Arg	TM2
NOX2	Met65Arg	TM2
NOX2	Leu66Pro/Arg	TM2
NOX2	Arg91Leu	Loop B*
NOX2	His101Tyr/Arg/Asp/Asn	TM3
NOX2	Met107Arg	TM3
NOX2	His111Arg	TM3
NOX2	Ser112Pro/Tyr	TM3
NOX2	His115Tyr/Gln/Asp	TM3
NOX2	His119Arg/Pro	TM3

NOX2	Leu120Pro	TM3
NOX2	Trp125Cys	TM3
NOX2	Cys126Arg	TM3
NOX2	Arg130Pro/Leu	Loop C
NOX2	Leu141Pro	Loop C
NOX2	Ser142Pro/Phe	Loop C
NOX2	Leu144Pro	Loop C
NOX2	Gly145Arg	Loop C
NOX2	Leu153Arg	Loop C
NOX2	Ala156Thr	Loop C
NOX2	Thr178Pro	TM4
NOX2	Gly179Arg/Glu	TM4
NOX2	Cys185Arg	TM4
NOX2	Ile187Arg	TM4
NOX2	Thr191Ser	TM4
NOX2	Ser193Pro/Phe	TM4
NOX2	Arg198Trp	Loop D
NOX2	Ser200Phe	Loop D
NOX2	Phe205Ile/Leu	TM5
NOX2	Thr208Arg, Thr503Ile	TM5
NOX2	His209Tyr/Arg/Gln/Asp	TM5
NOX2	Leu211Pro/Arg	TM5
NOX2	His222Asn/Tyr/Arg/Leu/Gln	TM5
NOX2	Ala224Gly	Loop E
NOX2	Glu225Val	Loop E

NOX2	Gln231Pro	Loop E
NOX2	Cys244Ser/Arg/Gly/Tyr	Loop E
NOX2	Gln246Pro	Loop E
NOX2	Cys257Arg/Ser	Loop E
NOX2	Pro260Arg	Loop E
NOX2	Gly275Asp	TM6
NOX2	Val295Glu	DHD*
NOX2	Lys299Asn	DHD*
NOX2	Thr302Pro	DHD*
NOX2	His303Asn/Tyr/Leu/Gln	DHD*
NOX2	Pro304Arg/Leu	DHD*
NOX2	Thr307Pro	DHD*
NOX2	Glu309Lys	DHD*
NOX2	Leu310Pro/Gln	DHD*
NOX2	Met312Lys/Arg	DHD*
NOX2	Gly322Glu/Arg	DHD*
NOX2	Ile325Phe	DHD*
NOX2	Val327Asp	DHD*
NOX2	Cys329Arg	DHD*
NOX2	Ser333Pro	DHD*
NOX2	His338Gln/Tyr/Asn/Arg/Asp	DHD*
NOX2	Pro339Leu/His	DHD*
NOX2	Phe340Ser	DHD*
NOX2	Thr341Ile/Lys	DHD*
NOX2	Leu342Gln	DHD*

NOX2	Thr343Pro/Ile	DHD*
NOX2	Ser344Pro/Phe	DHD*
NOX2	His354Pro/Arg	DHD*
NOX2	Ile355Asn	DHD*
NOX2	Arg356Pro	DHD*
NOX2	Gly359Val/Ala/Arg/	DHD*
NOX2	Trp361Arg/Gly/Leu/Cys	DHD*
NOX2	Thr362Arg/Lys/Ile	DHD*
NOX2	Leu365Pro/Arg	DHD*
NOX2	Cys369Arg	DHD*
NOX2	Asp378Gly	DHD*
NOX2	Pro383Leu	DHD*
NOX2	Lys384Asn	DHD*
NOX2	Ile385Arg	DHD*
NOX2	Gly389Val/Glu/Ala/Arg	DHD*
NOX2	Pro390Leu/Arg	DHD*
NOX2	Met405Arg	DHD*
NOX2	Gly408Glu/Arg	DHD*
NOX2	Ala409Glu	DHD*
NOX2	Ile411Phe	DHD*
NOX2	Gly412Glu/Arg/Val	DHD*
NOX2	Thr414Ile	DHD*
NOX2	Pro415Arg/Leu/His	DHD*
NOX2	Ser418Tyr/Phe	DHD*
NOX2	Leu420Pro	DHD*

NOX2	Ser422Pro	DHD*
NOX2	Ala431Thr	DHD*
NOX2	Ile439Val	DHD*
NOX2	Cys445Arg	DHD*
NOX2	Trp453Arg	DHD*
NOX2	Asp456Asn	DHD*
NOX2	Gly472Ser	DHD*
NOX2	Leu474Arg	DHD*
NOX2	Thr481Pro	DHD*
NOX2	Trp483Arg	DHD*
NOX2	Gln487His	DHD*
NOX2	Ala488Asp	DHD*
NOX2	His495Pro	DHD*
NOX2	Asp500Tyr/His/Asn/Gly/Val/Glu	DHD*
NOX2	Thr503Lys/Ile	DHD*
NOX2	Leu505Arg/Pro	DHD*
NOX2	Thr509Asn	DHD*
NOX2	Tyr511Asp	DHD*
NOX2	Trp516Arg/Cys	DHD*
NOX2	Pro528Arg	DHD*
NOX2	Val534Asp	DHD*
NOX2	Cys537Arg	DHD*
NOX2	Gly538Arg/Glu	DHD*
NOX2	Leu542Ser	DHD*
NOX2	Leu546Arg/Pro	DHD*

NOX2	Glu568Lys	DHD*

Supplementary table 2. Reported missense mutations in NOX2 and p22 that cause chronic granulomatous disease. Asterisks represent mutations in regions that have not been modeled in the NOX2 structure.

Additional supplementary information – Uncropped SDS-PAGE, FACS gating strategy

Uncropped SDS PAGE from Supplementary Figure 1b

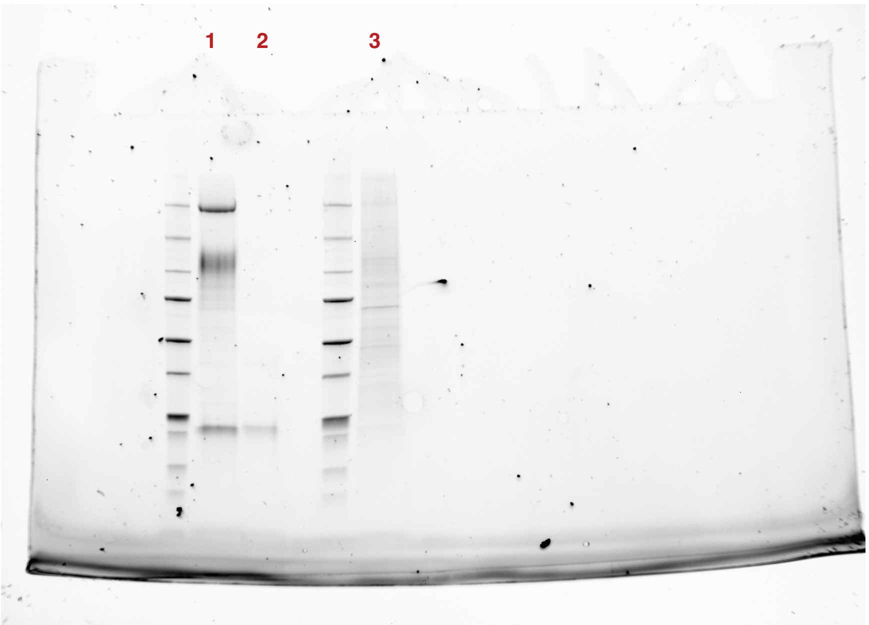

1 - Main peak - cryo-EM sample
2 - Second peak (excess 7G5 Fab)
3 - Unrelated sample - not part of NOX2-7G5 purification

FACS gating - Supplementary Figure 8a

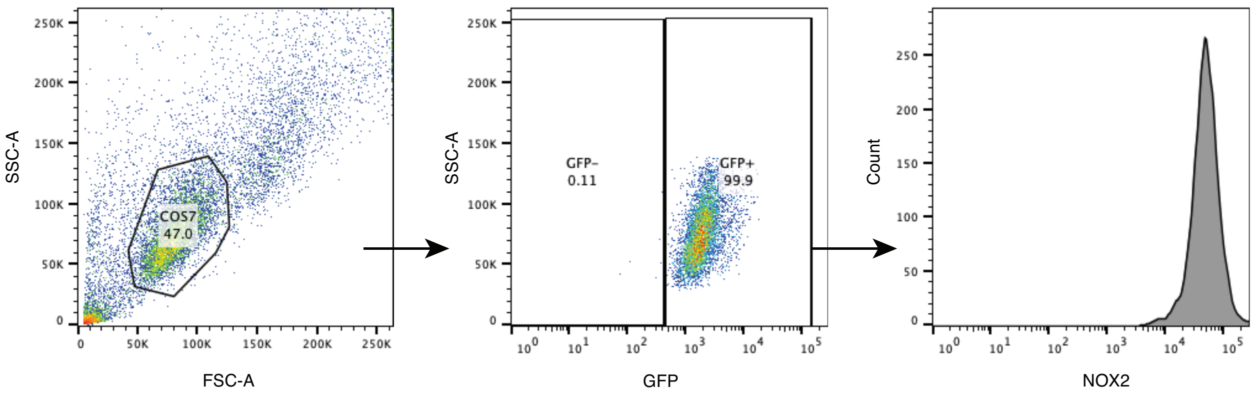

FACS gating - Supplementary Figure 9f

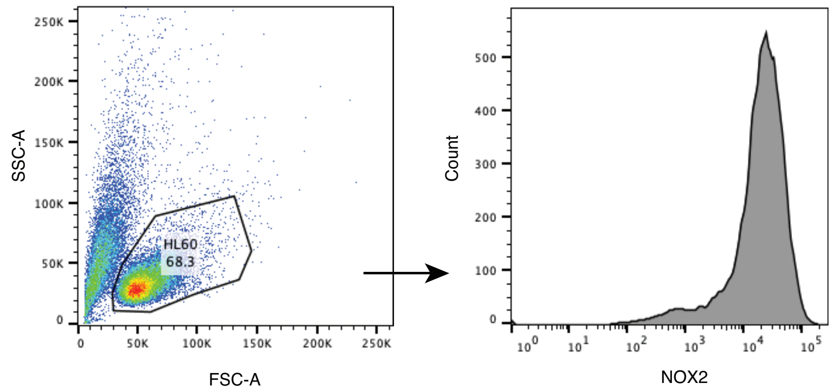

Supplement: Supplementary file 1 — Supplementary Information [file 41467_2022_33711_MOESM1_ESM.pdf]
