# Supplementary material for: Structure of the core human NADPH oxidase NOX2
Source: Nat Commun. 2022 Oct 14;13:6079. doi: 10.1038/s41467-022-33711-0 (PMC9568551; doi:10.1038/s41467-022-33711-0)
Supplement: Supplementary file 2 — Reporting Summary [file 41467_2022_33711_MOESM2_ESM.pdf]

nature portfolio

Corresponding author(s): Masureel, M., Payandeh, J., Yi, T., Koerber, J.T.

Last updated by author(s): Aug 26, 2022

Reporting Summary

Nature Portfolio wishes to improve the reproducibility of the work that we publish. This form provides structure for consistency and transparency in reporting. For further information on Nature Portfolio policies, see our Editorial Policies and the Editorial Policy Checklist.

Statistics

For all statistical analyses, confirm that the following items are present in the figure legend, table legend, main text, or Methods section.

n/a	Confirmed	
☐	☒	The exact sample size (*n*) for each experimental group/condition, given as a discrete number and unit of measurement
☐	☒	A statement on whether measurements were taken from distinct samples or whether the same sample was measured repeatedly
☐	☒	The statistical test(s) used AND whether they are one- or two-sided<br>*Only common tests should be described solely by name; describe more complex techniques in the Methods section.*
☒	☐	A description of all covariates tested
☐	☒	A description of any assumptions or corrections, such as tests of normality and adjustment for multiple comparisons
☐	☒	A full description of the statistical parameters including central tendency (e.g. means) or other basic estimates (e.g. regression coefficient) AND variation (e.g. standard deviation) or associated estimates of uncertainty (e.g. confidence intervals)
☐	☒	For null hypothesis testing, the test statistic (e.g. *F*, *t*, *r*) with confidence intervals, effect sizes, degrees of freedom and *P* value noted<br>*Give P values as exact values whenever suitable.*
☒	☐	For Bayesian analysis, information on the choice of priors and Markov chain Monte Carlo settings
☒	☐	For hierarchical and complex designs, identification of the appropriate level for tests and full reporting of outcomes
☒	☐	Estimates of effect sizes (e.g. Cohen's *d*, Pearson's *r*), indicating how they were calculated

Our web collection on statistics for biologists contains articles on many of the points above.

Software and code

Policy information about availability of computer code

Data collection	EPU 2.3.0.79REL
Data analysis	Prism 9 for MacOS, UCSF Chimera1.14, MapQ (v1.8.2), UCSF ChimeraX-1.2.5, ISOLDE1.2.1, cryoSPARC v3.1.0, Pymol 2.4.2, Coot 0.8.9.2, Phenix v1.19.2, MolProbity 4.5, FlowJo v.10.6.1 , FlowJo v.10.8.1

For manuscripts utilizing custom algorithms or software that are central to the research but not yet described in published literature, software must be made available to editors and reviewers. We strongly encourage code deposition in a community repository (e.g. GitHub). See the Nature Portfolio guidelines for submitting code & software for further information.

Data

Policy information about availability of data

All manuscripts must include a data availability statement. This statement should provide the following information, where applicable:

- Accession codes, unique identifiers, or web links for publicly available datasets
- A description of any restrictions on data availability
- For clinical datasets or third party data, please ensure that the statement adheres to our policy

All other data are included in the paper and the supplementary information files. Sequence information of NOX2 and p22 was obtained from the Uniprot data base (NOX2: P04839, p22: Q86SL0). Source data is provided for figure 4, and the following supplemental figures 1, 8, 9. The 3D cryo-EM density maps were deposited into the Electron Microscopy Data Bank (https://www.ebi.ac.uk/pdbe/emdb) under accession code EMD-26383. The coordinates were deposited in the PDB (https://www.rcsb.org) with accession code 7U8G.

Field-specific reporting

Please select the one below that is the best fit for your research. If you are not sure, read the appropriate sections before making your selection.

☒ Life sciences ☐ Behavioural & social sciences ☐ Ecological, evolutionary & environmental sciences

For a reference copy of the document with all sections, see nature.com/documents/nr-reporting-summary-flat.pdf

Life sciences study design

All studies must disclose on these points even when the disclosure is negative.

Sample size	Sample sizes were not predetermined for this study. Sample size for cryo-EM data were determined by the availability of microscope time. All cell-based assays were performed in duplicates and repeated at least three times independently. The sample size for the cell-based assays were limited due to a time-sensitive experimental setup.
Data exclusions	No data were excluded from the analysis.
Replication	Cell-based measurements were performed in duplicate. Replication was successful.
Randomization	Randomization is not applicable for the experiments involving cryo-EM. For cell-based assays data was recorded in varying order, other types of randomization is not relevant to this study.
Blinding	Blinding is not relevant to this study.

Reporting for specific materials, systems and methods

We require information from authors about some types of materials, experimental systems and methods used in many studies. Here, indicate whether each material, system or method listed is relevant to your study. If you are not sure if a list item applies to your research, read the appropriate section before selecting a response.

Materials & experimental systems

n/a	Involved in the study	
☐	☒	Antibodies
☐	☒	Eukaryotic cell lines
☒	☐	Palaeontology and archaeology
☐	☒	Animals and other organisms
☐	☒	Human research participants
☒	☐	Clinical data
☒	☐	Dual use research of concern

Methods

n/a	Involved in the study	
☒	☐	ChIP-seq
☐	☒	Flow cytometry
☒	☐	MRI-based neuroimaging

Antibodies

Antibodies used	In-house antibody: anti-gp91(phox) (7G5, produced at Genentech), anti-gp120 (isotype control, produced at Genentech), anti-rabbit CD11b (MCA802GA, biorad), anti-rabbit T-lymphocyte (MCA800GA, biorad), anti-rabbit IgM (550938, BD bioscience)
Validation	Validation of the antibody 7G5 was performed in this study through biochemistry analysis, cryo-EM 3D reconstruction and cell-based and cell-free functional assays as described in Supplementary Figure 1 , 2, 8 and 9. Validation of the other antibodies (anti-rabbit CD11b (MCA802GA, biorad), anti-rabbit T-lymphocyte (MCA800GA, biorad), anti-rabbit IgM (550938, BD bioscience)) used in this study were done by manufacturer and the details of these validation efforts are described on the manufacturer's website.

Eukaryotic cell lines

Policy information about cell lines

Cell line source(s)	HEK293 (human embryonic kidney cells, Expi293F Invitrogen), HL60 (human promyeoloblasts, CCL-240), COS7(African green monkey kidney fibroblast-like cell line, CRL-1651).
Authentication	HEK293 and COS7 cell lines were not authenticated. Whole genome sequencing was performed internally on HL60 cell line.
Mycoplasma contamination	Each cell line were tested negative for mycoplasma contamination.
Commonly misidentified lines (See ICLAC register)	No commonly misidentified lines were applied.

Animals and other organisms

Policy information about studies involving animals; ARRIVE guidelines recommended for reporting animal research

Laboratory animals	New Zealand White (NZW) female rabbits (Charles River Laboratories, Hollister, CA), 6 months old.
Wild animals	The study did not involve wild animals.
Field-collected samples	No field-collected samples were used in this study.
Ethics oversight	Animals used in these studies were maintained in an Association for Assessment and Accreditation of Laboratory Animal Care (AAALAC)-accredited animal facility. All experiments were performed in compliance with Genentech's Institutional Animal Care and Use Committee (IACUC) and National Institutes of Health's Office of Laboratory Animal Welfare Guidelines. Approval of the study design was obtained from the Genentech IACUC prior to the start of this work.

Note that full information on the approval of the study protocol must also be provided in the manuscript.

Human research participants

Policy information about studies involving human research participants

Population characteristics	There was no restriction on donors, genotype, past or current diagnostics, or treatment category prior to requesting samples for this study. The covariate-relevant population characteristics of the human research participant samples used in this study were male, asian, ranging between 50-60 years old.
Recruitment	The participants were recruited by Samples4Science donor program at Genentech. No bias expected to be present for this type of study.
Ethics oversight	Peripheral blood samples from healthy male and female donors at least 18 years of age were kindly provided by the Samples4Science donor program at Genentech. Donors provided written informed consent and sample collection was approved by the Western Institutional Review Board.

Note that full information on the approval of the study protocol must also be provided in the manuscript.

Flow Cytometry

Plots

Confirm that:

☒ The axis labels state the marker and fluorochrome used (e.g. CD4-FITC).

☒ The axis scales are clearly visible. Include numbers along axes only for bottom left plot of group (a 'group' is an analysis of identical markers).

☒ All plots are contour plots with outliers or pseudocolor plots.

☒ A numerical value for number of cells or percentage (with statistics) is provided.

Methodology

Sample preparation	COS7 cells expressing NOX2 subunits, wild type and gp91-deficient differentiated HL60 cells were evaluated by staining with 7G5 IgG at 5 µg/mL in FACS buffer-1 (PBS with 2% FBS and 0.2% NaN3) at room temperature for 1 hr. After washing with FACS buffer-1 twice, the cells were resuspended in FACS buffer-1 containing the 2 µg/mL of APC-conjugated F(ab')2-goat anti-mouse IgG (Thermo Fisher Scientific) and incubated at room temperature for 30 min. After washing with FACS buffer-1 twice, the cells were resuspended in FACS buffer-1<br>HL60 cells were differentiated with 1.3% DMSO in complete RPMI1640 medium for 4 days. 1x105 cells suspended in FACS buffer-2 (PBS containing 0.5% BSA and 2 mM EDTA) containing 25% of human serum were incubated with various concentrations of Dylight 550 conjugated 7G5 Fab or 7G5 IgG, at 37°C in the presence of 0.1% NaN3 for 4 hrs (cell-based affinity assay) or with Biotin conjugated 7G5 Fab or unconjugated 7G5 IgG at 37°C in the presence or absence of 0.1% NaN3 for 1.5 hrs (internalization assay), then washed twice with FACS buffer-2. For incubation with biotin conjugated 7G5 Fab or unconjugated 7G5 IgG, the cells were further stained with Alexa Fluor 647 Streptavidin (Biolegend, 405237) or Alexa Fluor® 647 AffiniPure F(ab')$_2$ Fragment Goat Anti-Mouse IgG (Jackson Imm lab, 115-606-071, 1:500, for internalization assay) respectively, at 4°C for 15 min, then washed twice with FACS buffer-2. The cells were resuspended in FACS buffer-2 with propidium iodide (BD Biosciences 556463, 0.5 µg/ml) for analysis.
Instrument	BD FACSCelesta, LSRFortessa, or FACSSymphony (BD biosciences).
Software	FlowJo v.10.8.1
Cell population abundance	Almost 100% of the sample are shown.

Gating strategy

Live cell population was gated on FSC vs SSC plot, or by propidium iodide negative population.

☒ Tick this box to confirm that a figure exemplifying the gating strategy is provided in the Supplementary Information.
